# Spirit of the Foreign Periodicals, &c.

**Published:** 1841-07-01

**Authors:** 


### Spirit of the Foreign Periodicals, &c.

---

### M. Andral on the Modern Doctrine of Humoral Pathology.

The following extracts, from one of the Lectures on General Pathology and Therapeutics recently delivered by this illustrious physician at the Faculty of Medicine at Paris, will be read with interest at the present moment by all who take an interest in the progressive changes of their professional literature.

"The history of those humoral alterations, which is to occupy our attention during the present session, comprehends three distinct parts—the study of the opinions and of the facts which belong to former times; the study of our existing actual knowledge and information which has been derived from the researches of modern chemistry; and, lastly, the pointing out of the gaps which still remain to be filled up, and for doing which we require much fresh and extended examination. If we have followed the progress of medicine in past times, we cannot fail to be struck with the very great difference between ancient and modern *humorism*. From the time of the Greek philosophers down to the tenth century—a period which constitutes the *first epoch* of humorism—the various doctrines that were successively in vogue were the offspring of mere conjecture. The four humours of the body, the four constitutions or temperaments, the four elements of nature, &c. about which so much has been written, were purely hypothetical creations, devised for the purpose of explaining the cause of diseases, as well as the mode of their progress and termination. The reasoning, which led to the admission of these humoral changes, was not exact, and was suggested merely by the necessity of discovering some mode of accounting for the development of certain maladies, which have evidently their origin in a lesion of the principal fluids of the system. The ancient humorists committed the serious error of proceeding by mere reasoning and conjecture in their examination of these changes, and of losing sight almost entirely of experimental research.

In the *second epoch* of humorism, comprising the period from the sixteenth century to recent times, the chemists, by substituting for the notions of their predecessors certain ideas derived from experiments performed in the laboratory, made certainly some progress in science; but their theories, although more exact than those which preceded them, were always imperfect and often quite erroneous, in consequence of their authors mistaking the animal economy for a mere laboratory, and of their neglecting to examine experimentally the fluids of the body.

Modern humorism has sprung up from the observation of facts in the very face of a solidism, which, being unable to render an explanation of all the phenomena of disease by the changes in the solids of the body, has been obliged to seek for it in the changes of the fluids. When we attentively study the different phases, through which pathological anatomy has passed during the last 40 years, we are convinced that one of its most immediate consequences is this very study of the various changes that are apt to occur in the different fluids of the body. And is it not natural that, after having examined by all known means of investigation the physical modifications which organs experience in the course of disease, and finding that those means fail to render an explanation of the morbid phenomena, we should interrogate the condition of the fluids by means of processes which chemistry alone suggests? The microscope, also, is an instrument which assists us in penetrating the intimate structure of the humours, and the nature of their alterations."

. . . . . . "It must be admitted that modern humorism has hitherto made but little real progress. In respect to the alterations of the fluids we are very nearly about the same point as the Greek physicians were in the days of Hippocrates in reference to the alterations of the solids. The old Coan, in one of his writings, tells us that we must not neglect these alterations, just in the same manner as the solidist physicians have in recent times told us that we should take account of the changes which may occur in the fluids; but little more was done in either epoch of our science than the occasional announcement of such vague advice.

To impart a more vigorous impulse to this department of medical enquiry, we must commence and prosecute a series of researches similar to what have been done for the solids during the last 40 years. Pathological anatomy reveals to us the lesions which occur in the constitution of the organs of the body, and chemical analysis will do nearly the same for the fluids: it is in truth a sort of pathological anatomy.

By separating the different elements which compose them, and by ascertaining if, and to what extent, these elements are altered in quality and quantity, chemical analysis may do much to assist us in the investigation of many diseases. It is in this manner that we must proceed to detect the lesions which affect not only the complex tissues which form the different organs of the body, but also the elementary tissues and the ultimate living molecules which concur in the structure of the solids.

To establish this molecular pathological anatomy, so to speak, we must avail ourselves of the aid of the microscope, and of all the various means of analysis which physical and chemical science furnish us with.

In short, modern humorism is an offspring of modern pathological anatomy; for is it not apparent that, if we prosecute our researches into the composition of morbid structures more and more profoundly, we are necessarily led to appreciate those minute changes in the condition of the fluids as well as of the solids of the body—the examination of which is the very basis on which the new humoral doctrine is founded."

M. *Andral* closes his observations with these valuable remarks:—

"To derive any important benefits from the study of the new doctrine, to which we have alluded, it is absolutely necessary that it be cultivated by men who do not profess to be exclusive advocates either of humorism or of solidism, and who are satisfied to advance with slow but sure steps in the path of experimental enquiry. It belongs to the professor of general pathology to enter upon and follow out this difficult career. In truth, general pathology is the true arena, where the great questions respecting all morbid changes in the system should be brought forward and canvassed. It is to it that we must look for the filling up of the gaps in science, by summoning to its aid every new means of research, and by pointing out the proper direction in which these should be employed. In general pathology we expect to find at once the inventory of the actual state of science, the evocation of its past career, and the preparation for its future condition: such is the task of the professor who understands the duty required of him. It does not consist, as has been too long imagined, in the mere study of the symptoms, the signs, and the causes of diseases, &c. those generalities which have not even the advantage of embracing all the curious facts of which internal pathology is composed."—*Gazette Medicale.*

---

### DR. MANDL ON THE CHEMICAL ANALYSIS OF THE BLOOD IN A PATHOLOGICAL STATE.

Dr. *Mandl* has contributed three very elaborate papers to the Archives de Medicine, for the latter months of last year, on this very difficult but very important and interesting subject.

The *first* is occupied with a review of the numerous, and from their discordance often bewildering, statements of different chemists and physiologists as to the composition and the relative qualities of the most obvious constituents of the blood—the fibrine, the globules and the serum. There is still much contrariety of opinion on this subject, and we are far from having arrived at any recognised exactitude of knowledge. Perhaps the very attempts, which have been made of late years since chemistry has made so much progress, to obtain too minute analyses of the animal fluids, has retarded and impeded our progress in our physiological acquaintance with them, alike in a healthy and in a morbid condition. To be a useful animal chemist, the experimenter must be a practical physician at the same time; and we cannot expect to obtain any important results, unless the various changes, which are induced on the fluids by disease, are attentively examined and duly appreciated at the bed side of the patient, as well as afterwards in the laboratory. To select one out of many proofs that *mere* chemistry can do but little for humoral pathology, we may allude to the circumstance of the two principles, albumen and fibrine, being recognised to consist of the same chemical elements in nearly the same proportions, while at the same time every one knows how different are the phenomena which they exhibit in the chemistry of the living body. And if we wished to shew the discrepancy of authors on the relative proportions of the different elements of the blood during health, we have only to mention that, according to *Lecanu*, there are about three parts of fibrine in 1000 parts, whereas *Berzelius* estimates the proportion at less than one part, *Fourcroy* as varying from 1 to 4, *Davy* as 1½, *Nasse* as 2½, *Müller* at nearly 5 (in the blood of the ox), and MM. *Andral* and *Gavarret* at 3. Well therefore may Professor *Giacomini* assert in a recent paper (v. Gazette des Hôpitaux, No. 20, 1840) that "if we compare the numerous tables on the composition of the blood published in works on chemistry, we do not find two that are not at variance."

If from the chemical analysis we proceed to the physiological interpretation of that most common phenomenon, the coagulation of the blood, we find as much discordancy of opinion as on the relative proportion of the fibrine. Some have attributed it altogether to the blood being at rest, and have therefore ascribed its fluidity to the circumstance of it being constantly in motion during life. But how then shall we explain the fact of the entire mass remaining perfectly fluid within the vessels in some cases of sudden death, and of the contents of a vein or artery in any intermediate portion of the tube between two ligatures, and thus kept in a state of complete repose, not becoming coagulated?

Others have sought for a reason of the phenomenon in the refrigeration of the blood when it is drawn from the body. But numerous experiments have shewn that this idea is quite untenable; seeing that the process actually goes on more quickly if the temperature of the blood is kept to the heat of the living body, and that, on the other hand, it is considerably retarded if the temperature is much reduced. *Hewson* has even proved that the blood after being frozen and again thawed will coagulate, as usual, into a solid and a fluid portion.

Then as to the influence of the atmosphere on the process of coagulation, various experiments have been at different times made to ascertain the effects of withdrawing its pressure, by introducing a vessel full of fresh-drawn blood under the bell of an air-pump; but hitherto no satisfactory conclusions have been drawn from these experiments; different results having been obtained by different men.

Then again, it has been supposed by some that the coagulation of the blood out of the body is somehow connected with, if not dependent upon, the disengagement of gaseous elements from its mass. *Home* and *Scudamore* have, for example, attached much importance to the escape of carbonic acid; but Dr. *Davy* has subsequently denied its influence altogether; and indeed might we not well ask, how does the blood not every moment coagulate in the lungs, seeing that there is a constant evolution of this very gas during respiration?

Dr. *Mandl* sums up his observations on this subject in these words: "What then up to the present moment is the result of all the chemical experiments which have been made to explain the coagulation of the blood? None, absolutely none.

N 2

How are we to account for the blood, contained in the jugular vein of a dog, rabbit, remaining liquid for some hours in a portion of the vessel which has been separated from the rest by means of two ligatures, and subsequently removed from the body of the animal? The coagulation is retarded under such circumstances by cold. But, when the vein is opened, the blood coagulates in the course of two or three minutes. Now how are we to explain these phenomena? We must confess that we are utterly unable to do so. This experiment is quite conclusive against the idea that coagulation is owing to the blood being at rest, as well as against that which attributes it to the influence of refrigeration."

Dr. *Mandl* subsequently alludes to the influence of certain chemical substances, when mixed with fresh-drawn blood, in retarding the process of coagulation. These substances either precipitate the albumen and fibrine in the form of molecules which do not afterwards coalesce, or they hold these elements in chemical solution. For example, by means of sulphuric acid we may obtain a solution of blood; but we have no right to say that under such circumstances we have prevented the coagulation of the fibrine.

With one short extract, which seems to us to suggest a useful distinction, we shall close our remarks on this subject........ "In studying the phenomena of the coagulation of the blood," says Dr. *Mandl,* "we ought to distinguish two moments or circumstances which are very different from each other:—the first is the solidification, or precipitation of the dissolved fibrine; and the second is the contraction of this fibrine newly solidified. This contraction of the fibrine may take place in three or four minutes, or it may require several hours, according to the refrigeration, the evaporation, the force of the jet, the greater or less quantity of the blood drawn, the external temperature, the humidity of the air, &c. &c. Now, authors have always confounded this second moment, the contraction, with the first moment, the precipitation; and yet these two phenomena are as distinct from each other, as the precipitation of a salt and its crystallisation. When *Hunter* wished to explain the process of coagulation by attributing it to the contraction which is observed to take place in the fibrine as in muscular tissue, he used to say—this *must* contract, because it *does* contract; he thought only of the second act or moment of the process."........ We proceed now to offer some remarks

*On the Formation of the Buffy Coat.*

Without entering at all upon the disputes of different chemists as to the nature of the buffy coat, we may merely state that, for all practical purposes, it may be admitted that it is part of the fibrine, held in solution in the blood, and deposited free from any admixture of red globules. The simple and beautiful experiment of *Hewson* proves this very distinctly: by removing with a spoon the upper colourless layer of the blood, he observed that the buffy coat is quickly formed in this decanted serum.

If we now enquire, what is the cause of this deposition of part of the pure fibrine of the blood on the surface of the clot, in certain forms of disease, a great number of most estimable authors will at once answer, "a greater than usually slowness in the act of coagulation." But another set of authors, whose names are entitled to as much credit, assert the very contrary, and distinctly maintain that this process actually goes on more quickly than usual, when the buffy coat is formed. For example, *Andral* and *Forget,* in their recent memoirs, state; "the separation of the blood into its solid and fluid portions takes place rapidly in plethora and in inflammatory diseases, as daily observation testifies."

We cannot, therefore, assent to the opinion that the formation of the buffy coat is owing to a retardation in the process of coagulation. Neither can we ascribe it, as some have done, to a mere excess in the quantity of the fibrine existent in the blood; for it has been amply shewn by several writers that not unfrequently the blood contains an unusual quantity of this constituent, and yet no buffy coat is formed on its surface.

M. *Magendie*, in his recent work on the physical phenomena of life, endeavours to explain the difficulty by ascribing the buffy coat to "one of the most simple and best understood physical phenomena, the relative gravity of the globules and of the fibrine; the former having a greater specific gravity than the latter tend to fall to the bottom of the mass, and thus the fibrine collects, more or less exempt from them, at the surface."

This explanation may be correct; but certainly the premises on which it is founded have not been proved. We do not for example know for a certainty that there is any alteration in the specific gravities of the fibrine and of the globules; and we should also remember that it is part only of the fibrine that collects at the surface, while another part sinks down, and entangles in its meshes the globules.

M. *Mandl* proceeds to expound his own views on this question. The following extract will enable our readers to understand the scope of his researches.

...... "Every one is agreed in admitting that in the formation of the buffy coat the red globules are precipitated before the fibrine is coagulated. Now on what does the formation of this buffy coat depend? we answer on all those circumstances which tend to favour this precipitation of the globules before the coagulation of the fibrine. Among these circumstances we may first mention the specific gravity of the serum. Whatever renders the serum lighter than usual, may give rise to the formation of the buffy coat, since the globules are then precipitated more quickly than usual. Again the blood and the serum, when cooled, are more dense and heavy than when they are warmed; and, therefore, whatever tends to keep the blood either warm or to cause it to cool will have the effect of on the one hand favouring, and on the other hand of retarding, the formation of the buffy coat. By attending to these circumstances we can explain the results of numerous experiments which we find recorded in the writings of various authors, but which are otherwise not easily intelligible. The small interrupted stream from a small opening in a vein, the vessel into which the blood is received being shallow, cool, and held at a distance from the arm, a cool state of the atmosphere, &c. &c., are so many circumstances which are unfavourable to the *franche* formation of the buffy coat."

In illustration of his views Dr. *Mandl* states, that from various experiments we have reason to believe that the specific gravity of the blood in inflammatory diseases is, in spite of a greater quantity of fibrine contained in it than usual, somewhat lower than in health. He appeals to the writings of *Thackrah, Scudamore, Babington,* and *Davy,* in proof of this assertion. *Nasse* also, of *Bonn,* in his recent work—Ueber das Blut, 1836—distinctly states that the buffy coat is stronger in proportion as the specific gravity of the blood is less, and that it forms with much greater difficulty on blood that is very dense.

If it be now asked, to what is this diminution of the gravity of the blood in inflammatory diseases owing, M. *Denis* (Etudes Chimiques sur le Sang) tells us that there is, under such circumstances, a deficiency of the usual quantity of the albumen and of the saline matters dissolved in the serum. This statement has not, however, been confirmed by the subsequent researches of MM. *Andral* and *Gavarret,* nor by those of M. *Lecanu.*

It is not improbable that the specific gravity of the globules themselves may vary a good deal under different circumstances; but as yet we have no satisfactory data on this subject.

### *Of Blood deficient in Fibrine.*

In not a few diseases is the blood found in what has been called a dissolved state, more than usually liquid, its coagulum being soft and imperfectly formed, and its watery portion more than usually abundant. Such is the case in almost all malignant fevers, in scurvy, in purpura, &c. &c.

Let us consider the state of the blood in scurvy for a few minutes. It is admitted by all authors who have seen much of this disease, that there is not only a marked deficiency of its fibrine, but also a very considerable increase of the alkaline salts of the blood. Now what is the effect of these salts on the coagulation of healthy blood?—to retard, or even to prevent the process altogether. Dr. *Mandl* seems to think that we are not to infer, from this condition of the blood, that there is necessarily any considerable diminution in its normal proportion of fibrine, but only that this element is held in solution in such a manner that it is not easily precipitated from the other elements of the mass.

Our information is as yet very defective as to the existing proportions of the saline matters of the blood in those other diseases where the blood is known to be more than usually thin and diffluent. It is worthy of notice that the commixture of putrid animal matters with healthy blood has nearly the same effect upon it as the addition of a quantity of an alkaline salt—viz. to impede the coagulation and to cause the mass to remain more fluid and dissolved. Whether the blood becomes actually impregnated with any miasmatic particles in typhus and other malignant fevers is not easily determined; but certainly there is nothing improbable in the supposition.

"We regret," says Dr. *Mandl*, "that we do not possess more complete analyses of the blood in typhoid fever. Is the fibrine actually diminished in quantity? and is the proportion of the saline ingredients, or of the albumen, which in our opinion produces analogous effects, at all increased? As yet we do not accurately know; but it may be readily understood, from what we have previously stated, that we must be on our guard in at once concluding that there must necessarily be a diminution of the actual quantity of the fibrine merely from the amount of it obtainable from the coagulum. M. *Denis* positively attributes the defect of coagulability and the other differences of the blood in typhoid fever to an augmentation of its saline ingredients."

Dr. *Mandl* next alludes to the alleged changes of the globules of the blood in disease.

"We shall now," says he, "enquire how far circumstances may influence the proportion or relative quantity of the globules in our examinations of the blood, and shall allude more particularly to that condition of the blood, where the coagulum is found to be more than usually soft and unadherent, because it is precisely in such a case that experimenters have discovered an abnormal proportion of the globules.

To determine the proportion of the globules in any quantity of blood, we divide it into two parts; in one of which we ascertain the quantity of the fibrine by stirring it briskly with a bunch of rods, and then deduct the weight of the fibrine so obtained from the weight of the coagulum, in the other portion of blood previously dried. Now, the fibrine being often unusually soft, it will be found to be very difficult to separate it completely; and hence the proportion of the globules will often be estimated as considerably higher than is normal, in consequence of a portion of the fibrine remaining in union with them.

Again, we have previously seen that a firm and well-contracted coagulum contains a large proportion of globules, and that, as a certain quantity of them escape our analysis, the exact amount of this proportion is apt to be understated. On the contrary, in a coagulum which is soft and does not retain its globules so firmly, the proportion of these globules is apt to be estimated too highly. It results from our observations that the more quickly that the fibrine coagulates, the less will be the quantity of it (the fibrine) that is found united or associated with the globules; and, on the contrary, the more slowly that the fibrine coagulates, the larger will be the quantity of it that is found with the globules: in other words, the relative proportion of the globules will be diminished in the first instance, and increased in the second. Now the presence of saline matters, which retard the coagulation of the blood, must produce on the one hand a soft coagulum, and on the other hand, an apparent increase in the proportion of the globules."

MM. *Andral* and *Gavarret* have, in their recent memoirs on the changes of the blood in various diseases, characterised the second class in their humoral nosology, as that in which the proportion of the fibrine of the blood is often diminished while that of the globules is often increased. In this class they have arranged all the tribe of continued fevers upon what we consider to be very insufficient information. For other observers, as Dr. *Reid Clanny* and Dr. *Jennings,* have expressly stated that, according to their researches, there is a notable diminution in the proportion of the globules of the blood in these very diseases. The question therefore still remains undecided. Indeed much, very much, requires to be done before we can assure ourselves of having arrived at any exact conclusions on many of the apparently most simple questions connected with the physiological and pathological conditions of the blood."—*Archives Generales de Medicine.*

---

### Influence of the Specific Density of the Blood on the Formation of the Buffy Coat.

During the last and the present year, much attention has been paid by several French physiologists to the various circumstances which influence the formation of the buffy coat on the blood; and as a new humoral pathology, so to speak, is gradually establishing itself in medical literature, it is necessary to be aware of the various discoveries, real or imaginary, of those who engage in the analysis of the animal fluids. The name of M. *Donné* is well known to all who take an interest in such pursuits. He has already done much in the field of microscopic enquiry applied to the investigation of the texture and composition of many parts of the body, both in disease and in health, and he still pursues his researches with all the ardour of a genuine lover of science. At the beginning of the present year he read a paper before the Academy of Sciences on the coagulation of the blood. His main object was to point out the conditions of this fluid, which favor the formation of what is called the buffy coat on its surface. Whenever the red globules are deposited more quickly than the fibrine, this phenomenon is induced. Now this may arise from two causes—either a more than usually slow coagulation of the blood, or a state of inferior specific gravity of this fluid. The former of these states has been long known and attended to; whereas the influence of the latter has hitherto been scarcely ever noticed. Now the latter condition—that of diminished specific gravity—is generally produced by previous loss of blood; and hence it is usually observed after venesections have been practised to any considerable amount. The mass of the blood having become less tenacious and viscid, its red globules precipitate themselves with greater rapidity than usual; and thus there is a tendency to the formation of a super-posed stratum of fibrine on the surface. It is from having omitted to take these circumstances into account, that hitherto medical men have been puzzled to explain the formation of the buffy coat in certain cases, more especially in acute rheumatism, at the second or third bleedings, when the blood drawn at first had not exhibited any appearances of it. The cause of this is, that the blood had been at first too dense and tenacious to allow the easy deposition of the red globules; but no sooner was it rendered somewhat thinner by venesection and the use of other antiphlogistic remedies, than the globules subsided more quickly and the fibrine was thereby permitted to collect on the surface of the coagulum.

---

### M. Raciborski's Critical Remarks on Auscultation.

M. *Raciborski,* a most intelligent physician resident, we believe, in Paris, seems to have discovered (!) that, in spite of the many works published on this subject, and all the confident assurances of professed stethoscopists as to the almost infallible accuracy of auscultatory diagnosis, a vast deal of error and of uncertainty still prevail. Much of this error and uncertainty he attributes to the attempts made by so many writers to reduce the phenomena of auscultation to the unvarying accuracy of an exact science, by boldly announcing that the existence of certain of these phenomena most indubitably demonstrates the existence of certain morbid states.

"Scarcely," says he, "had *Laennec* taught us to distinguish some *rales* in the pathological states of the respiratory organs, and scarcely had we began to make ourselves acquainted with the relations of some of these *rales* with the diseases in question, than every new sound was regarded as a pathognomonic sign of a disease, and we were told that every affection of the lungs must have its particular sound, the existence of which alone, and by itself, was sufficient to indicate the presence of the particular morbid state. Hence has arisen the tendency to a continual augmentation of the auscultatory *bruits*. In the impossibility to increase the catalogue of the diseases of the lungs, each of which had received from *Laennec* a particular sound, it has been the custom of late years to divide each disease into several stages, awarding to each of these stages its (alleged) characteristic rale.

Thus we hear not only of *cavernous* but also of *cavernulous*, and not only of *crepitant* but also of *sub-crepitant rales*."

There is much truth in the following remarks on the *ultra-ism* of many professed stethoscopists.

"The great error which has been too often committed has arisen from the attempt to attach a too exclusive importance to the phenomena of auscultation, and from regarding it as a science of sounds, each of which is presumed to indicate a particular morbid state. Now we have no hesitation in boldly affirming that there is scarcely a single *rale* which has a determined and invariable value, and that it is almost impossible to predict from the mere existence of any one rale the name of the disease to which it corresponds. For example, I defy the most experienced auscultator to distinguish, with his eyes closed, and without putting any questions to the patient or his friends, the greater number of cases of incipient tubercles from pneumonia in the first degree, or from œdema of the lungs, from hæmoptysis, or even from pulmonary emphysema (!) Again the sibilant *rale*, or the bass-string *rale* does not indicate the existence of pulmonary hepatisation more than it does that of pleuritic effusion; and even the gurgling *rale*, which is usually regarded as a pathognomonic sign of tuberculous evacuations, may be heard where there is no other morbid change but a mere dilatation of the bronchi; and lastly, the metallic tinkling is known to be present in cases of effusion into the pleura, as well as in those of pulmonary excavations."

M. *Raciborski* examines the separate *rales*, described by *Laennec* and others, commenting on their value as he goes along.

As to the *crepitant rales*, which is so generally admitted as characteristic of the first stage of pneumonia, he says that not only is this sound heard in other diseases, as in bronchitis and tubercles, as well as in inflammation of the lungs, but also that in the commencement of this very disease we not unfrequently discover a *sub-crepitant rale*—alleged to appertain almost exclusively to bronchitis or to pulmonary œdema—or even a *mucous* one.

He does not wish, he says, to undervalue the importance of auscultation, but only to caution his readers against the exaggerated statements of its value by its over-enthusiastic disciples, and to impress upon them the necessity of, in all cases, associating its results with those derived from other means of diagnosis. One, certainly, of the most valuable of these means is percussion—which, when skilfully performed, will often tend to correct certain mistakes that are apt to be made if we trust too much to mere auscultatory phenomena, and on the other hand, will greatly confirm the value, when their results happen to coincide, that we are to attach to these phenomena.

We cannot, as a matter of course, follow M. *Raciborski* in his very judicious examination of the signs furnished by percussion for the diagnosis of chest complaints, and must limit ourselves to the selection of a few extracts.

How to distinguish pulmonary hepatisation from effusion into the pleura.

"When the dullness of sound occupies the lower part of the chest, it is more difficult to determine at first, if this be owing to compression of the lung from effusion, or to an induration of the pulmonary tissue. In the majority of cases, however, we have only to attend to the *expression of the disease*, to be able to discriminate these two morbid states. The absence of expectoration in the former case will very often suffice to determine this point; and, if this will not do, there are other circumstances which will much assist our diagnosis. Thus in pleurisy the degree of dyspnœa is rarely in proportion to the extent over which the dullness on percussion is perceived: we often meet with cases where there is very considerable pleuritic effusion, although the respiration is by no means much distressed; whereas, in pneumonia, the degree of the dyspnœa is usually greater than the extent of the dullness might lead us to anticipate. Again, when the pleuritic effusion is considerable, there is usually a perceptible enlargement or expansion of the affected side; and if the hand be applied to the side while the patient speaks, the vibrations of the voice are scarcely, or not at all, perceptible."

M. *Raciborski* dwells with much emphasis on the value of the dullness on percussion over the upper part of the chest, in a patient who has had a cough for some time, as a symptom of tubercular induration.

...... "As this dullness does not depend so much on the presence of the tubercles themselves, as on the secondary alterations of the pulmonic tissue occasioned by them, we cannot be surprised that we meet with the symptom occasionally at the very commencement of the tuberculous deposit, when neither the presumed number nor the volume of the tubercles might lead us to expect it. Thus the dull sound perceived over the apex of the lungs may indicate the presence of tubercles in a person who has had a cough only for a week or two—provided there be no reason to suspect at the time a pneumonia. On more than one occasion we have been able to detect the existence of a tuberculous affection in the course of measles, although the patients had had no cough before the invasion of the exanthem."

M. *Raciborski* very strongly recommends that in all suspected cases we should employ percussion, not only of the subclavicular regions but of the clavicles themselves. It is the very apex of the lungs that is usually the early seat of tubercles; and hence it is important to examine the chest as high up as possible.

He closes his remarks on percussion in the following words:—

"The study of percussion being incomparably more easy than that of auscultation, our readers will readily understand our motives in first alluding to it. As it is often quite sufficient by itself for the diagnosis of many thoracic diseases, we should always commence the examination of our patients with ascertaining the results afforded by it, before proceeding to the auscultation of the chest."

We proceed now to notice some auscultatory phenomena.

### *The Expiratory Bruit or Murmur.*

"The *bruit* of inspiration is in health always longer and stronger and therefore more audible than the *bruit* of expiration—this latter being occasionally not all recognisable.

Whenever the pulmonary parenchyma becomes more or less impermeable to the air, the expiratory *bruit* becomes more lengthened and acquires more of the character of the inspiratory one: sometimes indeed it exceeds the latter both in strength and in duration, when the pathological condition of the lungs is very extensive or considerable.

Now whether this impermeability be occasioned by induration of the pulmonary tissue, or by compression of it from fluid effused into the pleura, the expiratory murmur is found to be prolonged, so as either to equal or even to exceed the duration of the inspiratory murmur.

...... It follows from our researches, that the prolongation of the expiratory murmur is one of the most constant characters of a tuberculous affection of the lungs—at least when the morbid deposit has already excited those consecutive alterations of the pulmonic tissue, which tend to render it more or less impermeable to the air. Whatever tends to indurate and solidify this tissue, will be found to cause the expiratory murmur to be more *prononcée*. Hence this auscultatory phenomenon is always associated with a dullness on percussion over the affected part of the lungs.

The expiratory sound is usually very much prolonged when the bronchial *souffle*, or blowing sound, is heard."

M. *Raciborski* proceeds to comment on the different abnormal respiratory sounds, or *rales* as they have been called, which have been described by authors. He condemns the fashion of subdividing and multiplying these, which has been so zealously pursued by some.

For instance, M. *Fournet*, in his recent work, has described no fewer than *five* different varieties of the vesicular *rale*, according as it is modified in active congestion of the lungs, in pulmonary œdema, in acute capillary bronchitis, in pneumonia, and, lastly, in relapse of pneumonia. Now what good can such perplexing minuteness do, either to diagnosis or to treatment? Assuredly none. All *rales* may be conveniently arranged into two heads, according as they are produced by the formation of *bullæ* in the bronchi or pulmonary vesicles, or by the vibration of these structures, in consequence of a contraction of their usual dimensions. Hence there are two leading kinds of *rales*—the bullar and the vibratory.* Perhaps we should add to these, the *metallic tinkling sound*, in consequence of the characters which are peculiar to it alone. These three kinds of sound are all that need be attended to in the diagnosis of the diseases of the respiratory organs.

*The Bullar Rale.*

The varieties of this sound are very numerous, according to the size of the bronchi chiefly affected, the viscosity of the fluid secreted, the force of the inspired air, &c. Some varieties are much more *dry* and crackling than others; but all arise from the formation of bubbles caused by the displacement of a liquid.

...... "The dry or moist tone, which often distinguishes the varieties of the bullar rale, is only a secondary character, and is dependent on the degree of viscosity of the bronchial secretion. The more viscid that the secretion is, the drier is the sound produced, so that the large gurgling bubbles may give rise to a sound that is quite like the crackling of a piece of folded parchment. But however dry the sound may be, provided it has not a vibrating character, we may be assured that it arises from the formation of bullæ in the air passages."

The diagnosis of the various diseases, in which the different kinds of bullar *rale* are perceptible, is to be determined by the presence or absence of various collateral symptoms and conditions—such as the duration of the disease, the existence or not of pain in any part, the dullness or not on percussion over the part where the bullar *rale* is heard, the character of the sputa, &c.

M. *Raciborski* confesses that it is sometimes extremely difficult, if not impossible, to discriminate, by any auscultatory symptoms, pneumonia from capillary bronchitis. Perhaps the best way is to ascertain the extent over which the bullar *rale* is heard: where this is limited the disease is most probably pneumonia; and

---

* The division of the *rales*, described by M. *Beau* in his late ingenious papers, is exactly the same—vide the last No. of the *Medico-Chirurgical Review*, p. 490.

on the contrary, where it is heard over a considerable extent, and still more if it be heard on both sides of the chest, we may pronounce the case to be one of bronchitis. Percussion, it must be confessed, will not much assist our diagnosis under such circumstances.

It is, however, in *chronic*, rather than in *acute*, diseases of the chest, that the existence of the *bullar rale* is valuable as a diagnostic symptom; and just because the *rational* symptoms of the existing disease are apt to be obscurely marked.

The chief chronic affections of the lungs, in which this *rale* is usually present, are tubercles, chronic bronchitis, emphysema, and œdema of the pulmonary tissue.

We have already, when alluding to percussion, pointed out the importance of attending to the part of the lungs where an abnormal sound is detected. This remark is equally applicable to the study of auscultation. If the *bullar rale* is heard over the upper part of one or both lungs, in a person who has had a cough for a considerable time, we have great reason to suspect the existence of tubercles*; and if to this symptom be added dullness on percussion of the clavicular region, the suspicion amounts almost to assurance.

In chronic bronchitis, on the other hand, the seat of the *rale* is usually towards the base of one or more frequently of both lungs, and moreover the resonance of the chest on percussion is, in this disease, little, if at all, impaired.

In emphysema of the lungs, also, the *bullar rale* is usually heard towards their bases; and the resonance of the chest on percussion is greater than in health.

In œdema of the lungs, on the other hand, the sound on percussion is generally more or less dull over a considerable extent; but the auscultatory symptoms are nearly the same as those heard in chronic bronchitis, and we are led to suspect the œdematous state of the respiratory organs chiefly from the co-existence of watery effusion in other parts, as in the limbs, abdomen, &c.

### *The Vibratory Rale.*

Under this general term, M. *Raciborski* comprehends the *sonorous*, the *sibilant*, the *snoring*, the *booming*, the *cooing*, the *chirping*, and the *rubbing rales* of *Laennec* and other authors. They have all the common character of communicating a vibratory or tremulous sensation to the hand applied to the parietes of that part of the chest where they are heard, and of not conveying the idea of the bursting of bubbles of air in a fluid.

"If," says our author, "it has been easy to prove that the *bullar rale* has but little value as a means of diagnosis, when regarded by itself and apart from other symptoms, it will not be difficult to shew that the *vibratory rale*, viewed in the same manner, is of even less value and less practical importance. As to the cause or *rationale* of this *rale*, if we except one variety—that which depends on the rubbing of false membranes on the surface of the pleuræ—we may say that all its forms or shades are caused by the vibrations of the air in the trachea or bronchi. In most cases these vibrations are owing to the flapping backwards and forwards

---

* M. *Raciborski* very truly remarks: "When the tuberculous affection is incipient, the bubbles of the *bullar rale* are very small, and not readily discovered on a superficial auscultation. We should never fail to make the patient cough occasionally, and attentively examine the upper part of the chest during and after the efforts of coughing. Sometimes it is only after repeated efforts, that we can detect the existence of the *bullar rale*, the sound of which is much like to that caused by pressing the saliva against the teeth, when the lips are closed."

He strongly urges the importance of auscultating the supra-clavicular, as well as the sub-clavicular regions, in all doubtful cases, assuring us that on several occasions he has detected the existence of tubercles by discovering a *bullar rale* in the former, while none was audible in the latter region.

of a small portion of viscid matter attached to some part or parts of the air-tubes. But whether this viscid matter be of the nature of mucus, or pus, or blood, or softened tubercle, we cannot determine from the sound emitted. The *rale* indicates nothing more than the existence of a certain morbid process in the bronchi; it is only by attention to the development and progress of the disease, and by other concomitant symptoms, including those discovered on percussion, as well as by the absence or presence of the *bullar rale*, that we can hope to ascertain its true nature."

The following remarks on the *retrograde resonance* of an abnormal sound along the bronchial tubes will remind our readers of the views of M. *Beau* on many of the phenomena of auscultation—*vide* the last two numbers of the *Medico-Chirurgical Review*, Foreign Periscope.

"A portion of mucus," he says, "attached to the superior part of one of the principal bronchi, may give rise to vibrations which will be perceived over the entire extent of the chest, below as well as above the seat of attachment. In such a case, not only can we not distinguish the locality of the disease, but we are often puzzled to conjecture its very nature."

*The Metallic Tinkling Sound.*

"This sound is a variety of the vibratory *rale*, and consists in sonorous vibrations, which resemble the last tones of a bell, or the sound produced by a fly, which, when buzzing to and fro in a porcelain vessel, causes the side of the vessel to vibrate. Similar vibrations may be produced by the inspired air, after having traversed a pulmonary fistula, entering the cavity of the pleura,—which (the cavity) is enlarged by the subsidence or recession of the lung in consequence of the greater or less quantity of fluid which may happen to have become effused.

The greater the force with which the air penetrates into the pleural cavity, the more distinct will be the *metallic tinkling:* hence it is generally stronger during the act of coughing, or when the patient speaks. The distinctness of this sound will be still more increased, if, instead of being caused by the mere admission of the air, it results from the resonance of another auscultatory sound which has a metallic character, and which is induced by the concussion of the molecules of a liquid against each other, as will often take place during the shaking of the chest by fits of coughing in cases of pleuritic effusion, or of immense vomicæ partially filled with fluid, or when we practise auscultation immediately upon the patient raising himself from the horizontal position."

From these remarks it appears that M. *Raciborski* groups together the *amphoric respiration* of *Laennec* with the sound which we are at present considering—the principal character of both consisting, he says, in a silvery or metallic resonance. Now this *resonance* is found to accompany the sounds produced either by the entrance of the respired air into a cavity of the pleura, or by the gentle succussion of the surface of an effused fluid, or by the fall of a drop of the fluid from the upper part of the cavity upon the fluid below. Hence the sound of the metallic tinkling appertains almost exclusively to hydro-pneumo-thorax, and to extensive tuberculous excavations.

M. *Raciborski* does not attach much importance to the auscultatory signs furnished by the voice—as bronchophony, pectoriloquy, and œgophony—unless they are in accordance with the results obtained by percussion and by auscultation of the breathing. He utterly rejects the idea of admitting the recently proposed auscultatory phenomenon, which has been termed *autophony*, and which consists in the (alleged) modification in the tone of the auscultator's own voice reverberated from the patient's chest. He adduces the testimony of M. *Bouillaud*, and of MM. *Barth* and *Roger* in confirmation of his estimate of its utter valuelessness. He closes his memoir by expressing his hope that he has succeeded in shewing that "the sounds of auscultation are far from having an equal importance in practice, and also that the most important of these sounds may be limited to a small number, which are easily recognised and distinguished from each other for all the purposes of useful diagnosis."—*L'Experience,* Nos. 176 and 177.

---

### M. Piorry on the Double Tic-tac Sound in the Aorta.

It is almost unnecessary to remind our readers that the name of M. *Piorry* is, more than that of any other writer, associated with the history of percussion, as applied to the discrimination of internal diseases. Within the last few months he has written an elaborate memoir, to prove that the dimensions not only of the aorta, in its ascending portion and its arch, but even of the pulmonary artery, may be most accurately determined by *plessimetric* measurements! How far other physicians will be made converts to his statements, and will be willing to admit their practical importance, remains to be seen;—for ourselves, we must confess that we have no great faith in the value of those attempts, however ingenious, to detect all the minute shades of alleged difference in auscultatory phenomena. If we are to believe M. *Piorry,* there is little difficulty in tracing out on the walls of the thorax the *exact* position of the different parts of the heart, and of the great vessels which issue from it, and consequently in detecting any enlargement of their volume by means of percussion alone. We much fear that but few medical men can ever hope to acquire such delicacy of perception, whether of hearing or of touch, to draw any practical utility from our author's instructions.

Passing over, therefore, the rules which he gives for his *plessimetric* investigations, we wish to draw our readers' attention to some remarks which he makes on the tictac or double sound perceived over the aorta.

............ "It follows as the result of a large majority of my observations, that the sounds perceived on auscultation over the aorta and the pulmonary artery are double, and not single, as supposed by *Laennec.* At first I enquired if the second sound or period of the tictac, observed in the large vessels which issue from the base of the heart, might not be produced in the pulmonary artery, while the first sound is produced in the aorta. But as I found the double beat over the aorta considerably above the point where it is separated from the pulmonary artery, I am now of opinion that we must admit that it is the seat of both sounds. However this may be, the fact is certain that in the majority of cases a double sound may be heard over the ascending portion of the arch of the aorta, and that very rarely indeed is one sound only audible......... Now where is the point at which this double or tictac sound ceases to be heard along the course of the aorta? For we certainly know that in the abdominal portion of the vessel we no longer perceive any double beats, and that in aneurysms of the descending aorta, which are accessible from behind to accurate investigation, we never hear any double beats, except when the tumor is in contact with the heart."......

We are certainly not satisfied from these statements of M. *Piorry* that there is any double sound from the aorta, unless what may be transmitted along it from the heart; and some of his own remarks appear to us in favour of this opinion. For example, he says: "several of the facts, which we have recorded, concur to shew that the sounds of the heart extend along the aorta. Thus in the 29th and 30th cases, a *bruit de souffle* was found to be propagated for some distance over where the *plessimetry* had shewn the arch of the vessel to be situated. Nothing similar is heard in pericarditis; the sounds in this disease being perceptible only over the spot from which they proceed."

M. *Piorry* alludes to the possibility of distinguishing a contraction of the orifice of the pulmonary artery from one of that of the aorta, by the greater limitation or restriction of the abnormal sound in the one case, and by its extension along the arch of the aorta in the other. This may probably be quite true; but how often will the distinction be appreciable?—that is the question for the practical physician.—*Archives Generales.*

### ON THE TREATMENT OF HÆMOPTYSIS WITH TARTRATE OF ANTIMONY.

It might at first be presumed that the Tartrate of Antimony, in consequence of its vomitive action, should rather aggravate than arrest the spitting of blood from the lungs; but, from the observations of *Stoll*, it appears that this is really not the case. The following instance, among many others, related by the illustrious physician of Vienna, is well worthy of notice.

"I remember," says he, "to have had under my care a young Turk who had been seized with bilious fever and with profuse hæmoptysis. I immediately ordered him a vomit. The attendants were frightened at the prescription, and most anxiously awaited the effects of the medicine; being afraid that the hæmorrhage would be greatly increased by the effects of vomiting. But, no sooner had the patient rejected a large quantity of bile from his stomach than all traces of the hæmorrhage ceased, and he recovered without any return of it."

After citing numerous similar cases, *Stoll* remarks:—"*Vomentes ne guttam sanguinis rejecerunt, quasi ipsa emesis hiantia pulmonum vasa quovis auxilio citius atque efficacius stringeret; et, vomitu jam peracto, aut nihil omnino sanguinis, aut ejus nonnisi paucum quid per intervalla et ad exiguum tempus comparuit.*"

It should be stated that *Stoll* seems to have employed this mode of treatment only in cases of hæmoptysis accompanied with bilious symptoms, and seldom in those where there was any marked predisposition to pulmonary consumption.

Several writers subsequently have adopted the emetic treatment of hæmoptysis, and very different results seem to have been obtained in the hands of different physicians; for, while some strongly approve of it, others as energetically condemn it. As a general remark, we may state that ipecacuan has usually been preferred as an emetic in cases of pulmonary hæmorrhage to any of the preparations of antimony; the former drug being supposed to possess a peculiar astringent property on the vessels of the lungs.

M. *Nonat*, one of the physicians of the Hôtel Dieu at Paris, however, has of late been using the tartrate of antimony in numerous cases of the disease, and reports most favorably of its effects. He has used it not only when the stomach and liver were evidently at fault at the same time, but also in many instances where there was no disturbance of the digestive organs, and where, therefore, the only indication was to arrest the discharge of blood from the chest.

*Case.*—A man, 30 years of age, was admitted into the Hôtel Dieu on the third day of an attack of hæmoptysis, which had resisted bleeding from the arm and the use of other remedial means: it was the first attack of the disease which he had ever experienced.

The symptoms on his admission were well marked: frequent cough, with the expectoration of frothy sanguineous sputa; mucous and sub-crepitant *rale* over the summit of the right lung (percussion gave a dull sound at this spot); sense of heat in the chest, and dyspnœa; pulse frequent and rather strong. There were also symptoms of considerable gastric disturbance, such as a coated tongue, bitter taste in the mouth, nausea, &c.

An emetic of tartrate of antimony was ordered; the stomach symptoms were quickly relieved, and the spitting of the blood entirely ceased.

The *second* case is very similar, and the *third* one was still more severe.

A middle-aged woman was seized with a second attack of hæmoptysis, which had already continued for four days before M. *Nonat* saw her. The previous attack had occurred two years before, and had lasted for nine days, before the hæmorrhage entirely ceased. An antimonial emetic was at once ordered her; it caused her to vomit three or four times freely; at the expiry of a few hours the sputa ceased to exhibit any trace of blood; and, from this time, they were nearly colourless.

After mentioning another successful case, M. *Nonat* then adduces the history of other two cases, in which the emetic practice failed altogether in arresting the sanguineous expectoration. In one of these, the patient was evidently labouring under tubercular phthisis in an advanced stage; the first dose of the emetic seemed to produce little or no effect on the pulmonary hæmorrhage; but, on being repeated a few days subsequently, it was distinctly increased, and considerable difficulty was experienced in subduing it.

M. *Nonat* candidly admits that the exhibition of emetics in a case of hæmoptysis, where we have reason to suspect ulceration of the lungs, is scarcely warrantable, and he very judiciously adds, that the practice is best suited to those cases of the disease, in which the hæmorrhage is of an active nature and is accompanied with the *molimen hæmorrhagicum.—Bulletin de Therapeutique.*

*Remarks.*—We have, for several years past, been in the habit of employing the tartrate of antimony in the treatment of hæmoptysis; but rather in small doses repeated at short intervals, than in full doses as an emetic. For example, a grain and a half, or two grains of the salt may be dissolved in eight ounces of water, and half an ounce of syrup of poppies added to the solution; of this mixture, two table-spoonsful should be given every two or three hours. We can confidently recommend this practice, both for its safety and its efficacy.—*Rev.*

---

### M. Trousseau on Tracheotomy in Chronic Diseases of the Larynx.

Professor *Trousseau* has now performed this operation in 113 cases—in eight only of which was it resorted to for chronic disease of the larynx. He ascribes the frequent failure of the operation to the too small size of the canulæ employed before the time of M. *Bretonneau,* his revered preceptor. He himself has recommended and introduced the use of a dilator, which much simplifies the operation, renders the ligature of the divided bloodvessels almost unnecessary, and greatly facilitates the introduction of the canula into the larynx.

But although the operation may be simplified, it must always be regarded as of serious moment, and should never be resorted to without absolute necessity. For, even when the death of the patient cannot be attributed to the operation, there is in many cases after its performance a marked tendency to the supervention of some acute disease of the lungs, which generally proves fatal.

Of the 8 cases, in which M. *Trousseau* performed tracheotomy in consequence of chronic diseases of the larynx, two died from rapid pneumonia. He attributes this tendency to pulmonic inflammation, which is apt to follow the performance of the operation, to the *stasis* or congestion of the blood induced by the embarrassment of the respiration. It would seem, however, from his own statements, that this predisposition to a rapidly fatal pneumonia is not greatest in those cases where the dyspnœa has been most severe before the operation, and moreover, that in such cases the relief obtained is often as decided and as permanent as in other instances, where the difficulty of breathing has not been so great. He has reported several cases in which he has performed the operation successfully on children when the asphyxia was already complete, and life seemed to be utterly extinct; and to these may be added a very extraordinary instance which occurred in the practice of M. *Bretonneau.* This distinguished practitioner being called to operate on a child, had the misfortune of seeing it expire, to all appearance, before the preliminary incisions were completed. M. *Bretonneau* however, without delay, opened the larynx, introduced a canula, and practised artificial respiration. At the end of a few minutes, the child began to breathe, and at length became completely resuscitated. A few days subsequently it was again seized with great difficulty of breathing; this, however, was ultimately relieved.

M. *Trousseau* relates a case which is very similar in many respects, to this one. In June, 1839, Count B. consulted him for a loss of his voice. His constitution had been a good deal injured by irregularities of living, as well as by syphilitic disease; and for the preceding four years he had been subject to epileptic fits. His voice, for a year or two before it was entirely lost, had been thick and indistinct. M. *Trousseau* was inclined at first to consider the *aphonia* to be owing to a chronic inflammation of the larynx, and recommended the local application of a solution of the nitrate of silver, and of the insufflation of a powder consisting of calomel and sugar, along with the use of antiphlogistic remedies, blisters to the throat, &c.

No relief however was derived from this mode of treatment; indeed the symptoms became worse. An active syphilitic treatment was therefore commenced; but in about a fortnight afterwards the patient was suddenly seized with a fit of suffocation. MM. *Marjolin* and *Cruveilhier* were called into consultation, and, as both these surgeons regarded the case as one of syphilitic disease, the same remedies were continued, along with a bleeding from the arm, and the insertion of a seton in the neck. The following night and day, the symptoms of asphyxia were terrible, and, as the patient seemed every moment about to be suffocated, it was resolved to perform the operation of laryngotomy. Before, however, this could be done, the respiration had ceased, the pulsations of the heart could scarcely be felt or heard, and the patient had all the appearance of a dead man; no blood flowed from the wound, and when the canula was introduced, no respiratory movements followed.

By first compressing the chest with great force and pulling down the edges of the ribs, so as to empty the lungs of the air they contained, and then drawing them up, as in a deep inspiration, the air entered the canula with force. These manœuvres were continued for nearly ten minutes, before the pulse at the wrist could be felt. The artificial respiration was persevered in for fifteen minutes more; and then the action of the heart and arteries was fairly re-established. Ten minutes later, the face was slightly convulsed; and soon afterwards an attack of general eclampsia succeeded, during which the air was drawn in with great violence into the chest, and this was followed by a state of stupor. In an hour and a half after the operation, the patient recovered his consciousness. From this moment, everything went on well; the canula was changed twice every day, and the distress in breathing gradually became less and less. In the month of December (the operation had been performed in the middle of August) the patient appeared to be perfectly well, although he still wore the canula. At this period, the larynx was found to have acquired nearly double its usual volume, and the angle formed at the meeting of the two thyroid plates had become very obtuse. When the canula was closed, no air passed through the larynx from the throat. The diagnosis therefore was, that a tumor existed in the larynx, which would ultimately prove fatal. The deglutition began about this time to be impeded; and at length, in the following May, the poor patient died. A cancerous tumor was found on dissection.

The embarrassment in such cases is to determine what is the exact nature of the existent disease. M. *Trousseau*, however, lays it down as a maxim that the operation is never useless, unless there be serious co-existing disease in some other part of the body at the same time; and even then that the life of the patient may be prolonged, although we cannot hope permanently to recover our patient. Whenever, therefore, the diagnosis of such a complication is uncertain, he thinks that we should always give the patient the chance of benefit, by performing the operation.

M. *Trousseau* insists much on using canulæ of a larger size than are usually employed. He uses a middle-sized one at first, and gradually increases the size, until the air ceases to make almost any noise passing through it during a deep inspiration.—*Journal des Connoiss. Med. Chirurg. and La Gazette Medicale.*

---

### Utility of Musk in Pneumonia accompanied with Delirium.

When sanguineous and other evacuations have been carried to a great extent in pneumonia and other inflammatory diseases, delirium not unfrequently supervenes. In such cases Professor *Recamier*—one of the most practical physicians of the French school—has obtained the most gratifying results from the administration of musk in large doses. This practice has subsequently been adopted with equal success by many others. Dr. *Roché* has recently detailed several cases in illustration of its good effects, in a late No. of the Journal des Connoissances Medico-Chirurgicales. We give an abridged report of one.

A man, 54 years of age, was seized with all the symptoms of severe pneumonia, on the 20th April 1840 : he was bled profusely from the arm, and for the next two days he was much relieved. A state of great restlessness and of mental incoherence surpervened, and the urine was passed involuntarily. Leeches were applied to the head, sinapisms to the calves of the legs, and repeated doses of antimony were administered. But no relief was derived from these remedies : the pulse at this time was frequent, small and compressible. A grain of musk was ordered to be given every hour ; and soon the patient fell into a tranquil sleep. The somnolence continued for nearly twenty-four hours, and then the patient awoke much refreshed, but not at all aware of what had occurred during the last few days. The musk was continued in less frequent doses, and in the course of a day or two he was fairly convalescent. About two scruples of musk had been taken in the course of three days.—*Gazette Medicale.*

---

### On the Dangers of Paracentesis Thoracis, and the best means of obviating them.

Dr. *Regbard* of Lyons, author of the following observations, is strongly impressed with the opinion that the chief danger of making an opening into the cavity of the chest, for the purpose of giving exit to any effused fluid, arises from the admission of the external air—whereby the fluid, of whatever nature it may happen to be, becomes vitiated, and inflammation of the lining membranes is apt to be excited. While a student at the Hôtel Dieu in Lyons, in 1816, he had an opportunity of watching several cases, in which paracentesis thoracis had been performed, and he was then led to the conclusion that it is during the act of *inspiration* that the external air is sucked in into the cavity of the pleura. If the wound or opening into the chest be of large dimensions, it will be at once apparent that, during every act of *expiration*, the effused fluid is forced out in greater or less quantity, while it is equally obvious that, during every act of *inspiration*, the air is drawn from without inwards. Dr. *Reybard*, in subsequently performing the operation on the human subject, used the precaution of plugging the canula of the trocar during each inspiration, and of allowing the escape of the fluid only during the movements of expiration. Unfortunately, however, when nearly all the fluid was thus safely withdrawn, the patient was seized with a violent fit of coughing during an act of expiration, and air was admitted into the pleural cavity. A shivering came on in a few hours after the operation ; this was followed by all the usual symptoms of pleuritis, which proved fatal on the third day. Dr. R. attributed the disastrous issue entirely to the admission of air into the chest during the act of coughing. He determined therefore to employ some new expedient in the next case, which might occur in his practice, to obviate this serious evil.

For this purpose he fitted on to the open end of the canula a bladder, which was easily emptied of all air, before the opening was made into the pleura ; the effused fluid flowed into the bladder. But there was this inconvenience ; that if air escaped at the same time, as must be the case in cases of hydro-pneumo-thorax, then this air was apt to collect in the upper part of the bladder, and to be again sucked into the chest during inspiration. To prevent this inconvenience, it occurred to Dr. R. that it would be sufficient merely to divide the bladder, and to leave attached a portion of it, of about 81 *centimetres* in length, to the extremity of the canula. This portion of bladder or gut, or what is still better, a piece of gold-beaters' skin, well softened in water, then acted as a valve—during expiration the fluid and air from the chest escaped by separating its thin and yielding, but constantly contiguous, surfaces, whereas during inspiration, it was drawn in and puckered around the opening of the canula by the suction exercised from within.

Dr. R. with the use of this very simple apparatus has subsequently performed the operation of paracentesis thoracis several times, without ever incurring the risk of the external air finding its way into the cavity of the pleura. While the stream of fluid that escapes by the canula is uninterrupted, there is little or no risk of this mischief; but in a minute or two it will be observed that the stream becomes less and less continuous, and then that the discharge takes place only during the acts of expiration. It is at this time that the bladder-valve of our author will be found to be truly useful, in preventing the admission of the air during the acts of inspiration.

As to the mode of managing different cases of empyema, Dr. *Regbard* observes:—

"If the effusion be recent, we may evacuate at once the whole of the fluid in the chest, because the lung, which has not been long compressed, will probably be able to dilate itself and resume its normal volume, when the compressing force is withdrawn. If, on the contrary, it has existed for several months, it may be more judicious to discharge the contents only partially, so as to permit the compressed lung to regain its former dimensions by degrees. Under such circumstances, I am of opinion that we should leave the canula, provided with the membranous valve, in the wound, so as to give a constant drainage, so to speak, from the pleural cavity, and thus enable the lung to expand itself gradually. If we adopt this practice, it will be found better to perforate the chest by drilling a hole through one of the ribs, than merely to divide the soft parts in an intercostal space with a trocar or bistoury."

It would appear, however, from a subsequent part of his memoir, that M. *Regbard* prefers to withdraw the canula after the operation, and re-introduce it once or twice every 24 hours for several days successively, than to leave it constantly in the wound.—*Gazette Medicale.*

---

### ON HYDATIDS IN THE CHEST—REMARKABLE CASE, WITH OBSERVATIONS.

There is scarcely an organ or tissue of the body in which these parasitical formations have not been discovered.

They are very common, it is well known, in certain ruminant animals, as in cows and sheep. In the human body, they are usually small and numerous; but in the lower animals we more often find only one hydatid which has attained a large size. In them, as in man, the liver and the lungs seem to be the organs in which hydatidic formations are most frequently developed.

What is the cause of such curious intus-conceptions? M. *Cruveilhier*, in his most ingenious work on this subject, published a few years ago, suggested that they might be owing to the introduction of unassimilated living molecules or germs into the blood from the alimentary canal, and circulated along with the blood to every organ of the body, until they became arrested in some part, and there grew and multiplied. Whether we admit this hypothesis or not, we cannot hesitate to acknowledge, with M. *Cruveilhier*, that insufficient nourishment and a residence in a damp unwholesome locality have an incontestible influence in favoring the production of acephalocyst germs. M. *Dupuy*, the distinguished professor in the veterinary college of Alfort, has arrived at the same conclusions with M. *Cruveilhier*.

By keeping in memory these two ascertained facts—1, that the development of hydatids is promoted by imperfect alimentation, and 2, that the organs most frequently affected, the liver and the lungs, are the organs of *hœmatosis*, through which the whole or the greater part of the blood passes—we certainly make some progress in discovering the ætiology of these singular growths.

*Andral* has reported in his *Clinique* seven cases of pulmonary acephalocysts. In all of them there had been during life an almost constant dyspnœa, which, in some of the patients, amounted to decided orthopnœa. The same suffering was present in the four cases related in the work of *Cruveilhier*—all of them having been afflicted with difficulty of breathing, which in two cases became every now and then quite suffocative. Neither of these distinguished physicians had been able to form a just diagnosis of the disease during the life of their patients; as, with the exception of the dyspnœa, the other symptoms were not quite alike in any two cases. Thus in one of *Andral's* cases, the symptoms seemed to indicate the existence of tubercles, in another of pulmonary emphysema, and in a third of chronic pneumonia or pleurisy.

The following remarkable case, recorded by Dr. *Max. Simon*, will be read with interest :—

A middle-aged woman had enjoyed good health till the year 1837, when she became, she said, subject to complaints in the throat, and to some difficulty of breathing whenever she walked quickly, or went up stairs ; there was also some degree of fever, and a slight cough at the same time. All these symptoms, however, gradually declined, and she recovered her usual health.

In 1839, the symptoms returned with greater severity, and, notwithstanding the use of bleeding, blistering, &c., the dyspnœa became suddenly almost quite suffocative, the face being livid, the throat swollen, and the cellular substance at the upper part of the chest emphysematous. The poor creature could not speak, but pointed with her hand to the front of the throat, as if something there prevented her drawing her breath. Dr. *Simon* suspected a sudden invasion of œdema of the glottis. In spite of all the remedies that were used, the woman died in about forty-eight hours after the seizure.

*Dissection*.—The larynx and trachea exhibited no abnormal appearances. On opening the chest, however, there was discovered an immense pouch developed at the expense of the mediastina, and situated between the two lungs, which were in consequence much compressed. This pouch had been accidentally opened, and was, therefore, nearly empty when examined. It still, however, contained a great number of hydatids ; but a much greater number were found floating loose in the blood, which had escaped from the divided venæ cavæ into the cavity of the pleura.

*Remarks*.—A curious feature in this, and in such-like cases, is the intermittence of the symptoms, and often, too, the sudden invasion of danger, when there must have been a constant and very serious lesion of so vital an organ as the lungs, during a great length of time. The most conspicuous symptom in the present instance, as well as in those related by MM. *Andral* and *Cruveilhier*, was the dyspnœa ; and neither auscultation nor any other means of dignosis could detect to what cause this dyspnœa was attributable. It is rather from the absence of those symptoms which characterise the more common diseases of the lungs, while there is still more or less dyspnœa, than from the existence of any special symptoms, that we may be led occasionally to suspect such a disease as that of hydatids in the organs of respiration.

"An important information, which may throw great light on a diagnosis so difficult, but of which physicians almost always deprive themselves, in consequence of not weighing with sufficient attention the account which patients give of their previous symptoms and condition, is the expectoration of either entire hydatids or of simple debris, the nature of which, however, is usually sufficiently distinct to be at once recognised. Thus, in the case which we have briefly reported above, the parents of the patient told us that he had on more occasions than one expectorated sputa, which were like pieces of skin rolled upon each other, and that, after such expectoration, he had always been much relieved: at the time we thought nothing of their statement, and only remembered it when the hydatid cyst was laid open on dissection. Consider how much this circumstance might have aided us, not only in the diagnosis of the disease, but also in suggesting a mode of treating it! For example, we at once understand how utterly useless large bleedings must be in such a case, whereas the employment occasionally of powerful emetics might have assisted nature in dislodging the contents of the hydatidic sac."

M. *Simon* closes his remarks by alluding to another case, which had occurred in his practice, and in which the expectoration of the hydatids appeared to be considerably promoted by the inhalation of the vapour of æther; the patient, however, ultimately died from disease of the heart.—*Journal des Connoiss. Med. Chir.*

---

### ON HYDATIC TUMORS OF THE LIVER.

Although allusions to these morbid formations may be found in the writings of the older physicians, and even of Hippocrates and Aretæus, it has been only within the last few years that much attention has been paid to their history.

As might be expected, much obscurity hangs over the question of their ætiology, or, in other words, of the causes which give rise to them. Professor *Cruveilhier* has suggested an opinion, more ingenious perhaps than probable, which rests on the frequency of hydatids in the tissue of the liver, and on the circumstance that this viscus is the *aboutissement* of all the abdominal venous blood, and consequently must receive along with the blood various materials, and organic materials which are imperfectly elaborated and animalised: these, he supposes, being deposited either in granulations or in the cellular tissue, become at length capable of a separate and individual life. It does not appear, as far as we know, that age, sex, or constitution, has any influence on their *genesis*. Among some of the lower animals, indeed, it would seem that imperfect nourishment and a damp unwholesome locality are sufficient to cause the development of hydatids in great frequency; but in the majority of the cases in the human subject, which have been collected together by M. Barbier in his recent work (*De la Tumeur Hydatique du Foie*, Paris, 1840), and amount to thirty-four in number, there is certainly no evidence to shew that these agencies had had any influence.

The existence or absence of the cyst, and the number and size of the hydatids, are circumstances which are very variable, and cannot be ascribed to any appreciable phenomenon. As to size, the hydatic tumor has in some instances been found to weigh several pounds; and then, as to their number, M. *Barbier* has found that, in twenty out of the thirty-four cases alluded to, there was only one hydatic tumor, in ten, there were more than one, and in seven, similar tumors were found in other viscera.

The symptoms of hydatic tumor of the liver are referrible almost altogether to its action on this viscus, and on the adjacent organs—as, for example, pain in the right hypochondrium, jaundice, ascites, and œdema of the lower extremities, disturbance of the digestive functions, and occasionally, also, of the respiration, circulation, and nutrition, &c. There is, however, one which is peculiar to this affection, and has been described under the appellation of the *bruit hydatique*, heard on percussion of the tumor. It is a complex phenomenon, being partly a sort of fluid sound perceived by the ear, and partly a vibrating trembling felt by the finger which strikes. It requires much tact and a good deal of practice to discover its existence in any case: but, if once fairly ascertained, it is pathognomonic of the disease.

There is another means of dignosis which may always be resorted to, whenever there is any uncertainty—we allude to that of puncturing the tumor with an exploring needle.

M. *Recamier*, so distinguished for his admirable skill in diagnosis, has practised this method with success in numerous cases.

The progress and duration of hydatic tumors of the liver vary exceedingly in different cases. In some, the disease does not give rise to any distinct symptoms until the swelling has attained a considerable size; in others, pain and feverish irritation accompany its early development.

The modes of its termination, when left to itself, are various, and this, like many chronic diseases, often exhibits the resistance which the animal economy opposes to the progress of morbid action. Sometimes the tumor breaks outwardly; at other times it opens into some part of the alimentary canal, or, after causing a perforation of the diaphragm, it discharges its contents into the bronchi of the adjacent portion of the lungs.

As to the treatment of hydatic tumors of the liver, we need scarcely say that any internal remedies can have little or no action upon their development, further than as they may tend to improve the general health of the patient, and thus fortify nature in her sanatory efforts. Surgery alone offers resources which inspire any confidence, and in the hands of an able practitioner may conduce to the cure of the disease. M. *Recamier*, as already stated, has set the example, and shewn us how to act under such circumstances. When once he has ascertained, by means of puncturing with a small trocar, that an encysted tumor is really present, he makes an eschar over the most prominent part with the potassa fusa, and repeats the application, at longer or shorter intervals according to the circumstances of the case, until the entire thickness of the abdominal parietes at that point is destroyed. The object is in part to reach as near as possible to the cyst before opening it, and in part also to cause an adhesion between the opposite surfaces of the peritoneum over it, so as to prevent the effusion of its contents into the cavity of the abdomen, when it is opened either by a new application of the caustic or by a cutting instrument.

When the sac has been evacuated, there still remains an important condition to fulfil; viz., to prevent its internal surface, which is sometimes very extensive, from suppurating and thereby inducing an irritative fever. To avoid this accident, M. *Recamier* is in the habit of filling the sac immediately with tepid water: this he renews every day, gradually diminishing the quantity injected, as the cyst contracts and its cavity becomes lessened.

By adopting the course of treatment now recommended, this accomplished physician has succeeded in effecting a cure of the disease in a considerable number of cases. Dr. *Jobert* and some other practitioners have also tried it, and have obtained most satisfactory results from it. The reader will find the detailed reports of several of Dr. *Jobert's* cases in the Thesis of M. Barbier.

---

### Treatment of Enlargements of the Spleen.

Dr. *Bouyer*, who informs us that his experience in the treatment of agues and of their consequences has been extensive for a series of years, regards almost every case of enlargement of the spleen as attributable to a passive congestion or accumulation of blood in its spongy parenchyma, the result of a retarded or impeded state of the abdominal circulation. It is not therefore by depletory means, or by any form of antiphlogistic treatment, that we can expect to reduce the (wrongly called) hypertrophied condition of this viscus. On the contrary, it is on fortifying the general system and promoting at the same time greater activity in the transit of the blood through the abdominal viscera, that we are chiefly to rely. If any dregs of the intermittent fever continue, the quinine, or some other form of bark, must be exhibited freely and in large doses. As the fever abates, the doses are to be gradually lessened; but the use of the medicine in some form* must be continued for a length of time. Frictions of the left hypochondrium, either dry or with some gently stimulating application, should be daily used; and immediately after each friction, a flannel belt should be put rather firmly around the abdomen. Out-door exercise is generally very useful; and, if the patient is residing at the sea-coast, he should try the effects of warm salt baths.

It is unnecessary to say that the state of the intestinal and the urinary secretions must be carefully attended to. Much harm is however apt to be done by the excessive use of purgatives; they tend to weaken the bowels, and thus to enfeeble the abdominal circulation. Dr. *Bouyer* does not approve of drawing blood by cupping over the left hypochondrium, as recommended by Drs. *Levy* and *Nonat;* but he does not object to the use of dry-cupping, which may tend to invigorate and excite the transit of blood through the spleen and the adjacent viscera. If the enlargement does not yield to the use of the means now mentioned, Dr. B. very strongly recommends the application to the affected side of an emplast. ammoniaci cum hydrargyro, with which has been incorporated a scruple or half a drachm of quinine, not omitting the use of the abdominal belt, and of bark or quinine internally at the same time.

No progress in the relief of the local evil can be made, if the employment of constitutional means be not carefully persevered in. In cases of very long standing, and where the viscus has become exceedingly enlarged, we are not to expect to effect a complete reduction to its healthy dimensions; we may, however, almost uniformly succeed in discussing the swelling very considerably, if the constitution of the patient be moderately sound.—*Gazette Medicale.*

---

### M. BRESCHET ON HYDROPHOBIA.

It seems that, upwards of five and twenty years ago, M. *Breschet*, in conjunction with the late Baron *Dupuytren* and M. *Majendie*, undertook the work of investigating some of the most interesting topics connected with the history of this obscure and dreadful malady.

In addition to watching most attentively all the cases that were brought to the Hôtel Dieu at Paris, these distinguished physiologists performed numerous experiments on animals at the Royal Veterinary School at Alfort and elsewhere, with the view of determining the laws of the transmission of the hydrophobic virus from one animal to another. Their conjoint labours were interrupted by the political convulsions of the period, and were never afterwards resumed, at least with decided energy; but as some of the results of their researches are perhaps not generally known, M. *Breschet* has very wisely deemed it right to make them public, more especially as, of late years, doubts have been expressed by some writers as to the contagious nature of hydrophobia.

---

* The decoction and the vinous tincture of cinchona are often exceedingly useful towards the decline of the fever.

The disease very rarely, if ever, appears spontaneously in other animals but those of the dog tribe; and certainly there is no authenticated case of its spontaneous uncommunicated origin in the human species. It has been from not having established the difference between the mere nervous affections in which there may happen to be a dread of fluids and a difficulty of swallowing, &c. and communicated *rabies*, that some authors have confounded *hydrophobia* with genuine *rabies*, and have thus been led into the error of regarding the latter disease as occasionally of spontaneous origin.

M. *Breschet*, after examining this question at considerrble length, proceeds to narrate some interesting experiments.

*On the transmission of Rabies from Man to the lower Animals.*

In May 1813, a man, 24 years of age, was bit by a mad dog, which had attacked a number of persons, several of whom were taken into the Hôtel Dieu, and had the wounded parts cauterised with a hot iron. Three of these persons were still in the hospital, when our patient, *Surlu*, was admitted on the 18th of June, on the third day after the first manifestation of the symptoms of *rabies*. He had received three bites on his right heel, which had been ineffectually cauterised an hour after the accident with the butter of antimony. The wounds healed, and the man, free of all apprehensions of future mischief, had been living an irregular debauched life, when suddenly a change came over his whole character; he lost his usual vivacity, all his movements became quick, hurried and impatient; he was every now and then sitting down, and then rising up without any object or motive; at times he began to weep, and express his alarm that he should become mad. Next day he manifested a repugnance to liquids, and when offered to him to drink, he forced them away from him. The eager vivacity of his looks, the restlessness of his whole body, the frothy fluid which was constantly flowing from his mouth, or which he spat out, the pain and sense of constriction in the back of the throat, and the horror at all liquids, left no doubt as to the nature of the case. MM. *Dupuytren* and *Breschet* determined to try the effects of injecting a solution of opium into the circulation. For this purpose a vein was opened in the arm, and, by means of *Anel's* syringe, a solution of two *grammes* of the aqueous extract in a small quantity of water was thrown into it. A few instants afterwards a pleasing calm supervened, so that, in the course of about four hours subsequently, four grains of the same medicine were administered in the same manner. For some hours there was a remission of the chief symptoms. But this calm was only temporary; all his former distress returned; he was in a state of constant agitation and restlessness, and every now and then uttered the most dreadful screams. Next day, he was more exhausted, but still retained his consciousness most perfectly. Six grains of the opiate extract were again injected into the venous circulation, and again it produced very soothing effects; but the patient became more and more exhausted, and he died in the course of the night.

MM. *Breschet* and *Majendie* having collected a considerable quantity of the frothy saliva from the mouth of the patient, inoculated two middle-sized dogs with it, inserting it into a wound on the back of the animals. This was done on the 19th of June; and on the 27th of July, one of the dogs (the other having made its escape before it was secured) manifested the symptoms of decided *rabies*. It bit several other dogs which were exposed, and every one of these became subsequently mad. "We could," adds M. *Breschet*, "by means of inoculation, keep up the disease in a number of dogs successively, in order that we might have constantly a supply of the *rabic* virus at command to enable us to pursue our experiments."

It was observed that the disease developed itself most frequently in from the 20th to the 30th day after the bite; occasionally, however, it did not appear until the end of three months afterwards.

There was reason to believe, from the results of several experiments, that the disease is not so readily communicated from one dog to another, when the contagious principle, the saliva, has been transmitted through three or four animals successively. If this remark be confirmed, it would shew that the virus becomes less and less active by passing successively through the systems of several animals.

In a few instances there was in truth no hydrophobia present; for the animals drank with eagerness any fluid that was put before them. This circumstance alone suffices to shew that genuine *rabies* and mere *hydrophobia* are by no means the same morbid states.

Before quitting this part of our subject, we may state that as yet we know of no authenticated example of the disease having ever been communicated from one human being to another, although there is every reason to dread such an event, if the saliva were permitted to be introduced into the system. We may mention also that when the patient, whose case we have given, was under treatment, there was a good opportunity of testing how far mental emotions can, of themselves, give rise to the symptoms of genuine *rabies*. When poor *Surlu* was brought to the Hospital, it soon became known to all the inmates that he was there, and that his death was looked for every hour. Now among these inmates were three persons who had been bitten by the same dog as the patient; and they remained in the hospital for nearly two months afterwards; but fortunately, notwithstanding this long protracted agony of fear, they entirely escaped any threatening of hydrophobia.

*On the transmision of Rabies from Carnivorous to Herbivorous Animals.*

M. *Breschet* exposed a strong healthy donkey to a dog that was furiously mad; the dog bit it in several places. Three weeks afterwards the donkey exhibited all the symptoms of *rabies* in the highest degree. "I may even affirm," says our author, "that I have never seen any animal that was more furiously rabid, or that manifested a stronger disposition to bite: it tore its own skin in several places."

M. *Breschet* also inoculated two horses with the froth collected from the throat of rabid dogs, by means of a sponge at the end of a rod. Both animals were subsequently seized with all the symptoms of *rabies*, but neither of them was so furious as the poor donkey.

*On the transmission of the Disease by inoculation from Solipedous to Carnivorous Animals.*

M. *Breschet* took some of the saliva of the horses and the donkey, that were rabid, and introduced it under the skin of the back of several dogs, all of which became mad in from 25 to 40 days after the operation. As this experiment was made not on one dog only but on several, and with uniformly the same results, we cannot hesitate to admit that the disease is readily transmissible from herbivorous to carnivorous animals—a fact which has been denied by some veterinary surgeons.

The dogs, which had thus become mad, were allowed to bite other dogs; and these too became affected in the same degree.

M. *Breschet* has therefore no doubt in his own mind as to the transmissibility of the disease; but he candidly admits that other experimenters have failed in their attempts to inoculate carnivorous from herbivorous animals (vide Journal de Magendie, t. viii.). As, however, one *positive* fact is worth a cartload of *negative* ones, the opinion of our author must be assented to. He has not been able to determine whether the disease can be transmitted by inoculation from one herbivorous animal to another of the same tribe. Several veterinary surgeons have denied the possibility; but our author seems not to be quite satisfied on this point.

M. *Breschet*, in the course of his researches, endeavoured to ascertain the effects of the poisonous virus, the saliva of a rabid dog, on some of the *rodent* tribe of animals. He therefore inoculated several rabbits, guinea-pigs, &c.—the animals died, but without manifesting any of the symptoms which are characteristic of the genuine disease.

The same result attended his experiments on birds.*

Before concluding his remarks on the transmission of the virus of *rabies* from one animal to another, our author informs us that he has made several experiments on carnivorous as well as on herbivorous animals, with the view of ascertaining whether the disease can be communicated by applying the saliva of a mad animal to an unabraded mucous surface in another. For this purpose he introduced portions of sponge charged with the saliva, into the mouth and rectum of several animals; but on no occasion did any symptoms of contamination ensue.

These negative results agree with those obtained by *Fontana* in reference to the poison of the viper; but they are in opposition to what has been stated by *Enaux* and *Chaussier*, who inform us that they once saw a man affected with genuine *rabies* after having received on his lips the saliva of a mad dog.

M. *Breschet*, in conclusion, briefly indicates the principal morbid changes which he has observed in the bodies of men and of the lower animals which have died of *rabies*.

We might expect that the pharynx always exhibited some abnormal appearances; but such was far from being uniformly the case. In some cases, however, the isthmus of the fauces, the velum, the pharynx and œsophagus, presented a strongly-marked *teinte rosée*, bordering occasionally on a violet hue. A quantity of frothy mucosity, similar to that of the respiratory passages, covered the surface of those parts as far down as the commencement of the œsophagus.

There was no very obvious lesion of the circulatory organs, except perhaps the distention of the pulmonary capillaries with black blood. We regret that we had not examined the blood either by chemical analysis or with the microscope: it is an omission which our author wishes to be filled up, although he has no reason to believe that the fluids have undergone any considerable change.

The examination of the nervous system has frequently revealed a highly-injected state of the vascular network of the pia mater, more particularly around the fissure of Silvius: in some cases a sero-gelatiniform effusion has been found in the cellular tissue of this membrane, and along the course of the principal arterial branches. It has been alleged by some, that there are usually observed signs of inflammation of the encephalon and of the spinal medulla; but, with the exception of a vascular state of the arachnoid membrane, and occasionally of a soft-

---

* M. *Breschet* remarks that the absorption of poisonous substances is usually very rapid and active in birds, and he mentions a curious circumstance which deserves to be generally known, as it may possibly suggest a useful hint in the treatment of poisoning with certain substances. After stating that he had performed a good number of experiments by inoculating various birds with the virus of some West India reptiles, and that they (the birds) all quickly died, unless prompt relief was given them—he adds—"I say unless prompt relief was given them; because we possessed a certain means of annihilating the deleterious action of the poison, which invariably succeeded, if not delayed too long. This consisted in establishing an electrical current from the wound of inoculation, by means of a metallic wire communicating with one of the poles of a galvanic pile in action, the other end of the wire being in contact with another part of the animal's body. Under the influence of the electrical current, the morbid actions induced by the poison became gradually less and less, until they finally ceased."

M. *Breschet* suggests the trial of it in cases of inoculation with the *rabic* virus.

ened state of some part of the cerebro-spinal axis, as well as of a sero-albuminous infiltration in the loose sub-arachnoid tissue, we have never detected any well-marked lesion to indicate a previous inflammatory action.

The lungs have been observed to exhibit a more or less deep red hue, which in some instances passed into a dark brown colour. This appearance was usually circumscribed to certain parts; more rarely it extended over the entire lungs. One of the most frequent morbid phenomena was the discolouration of the mucous membrane of the bronchi and occasionally also of the trachea: the hue was usually a dark red, verging into a violet or even a brown colour. In several instances there was interlobular emphysema of the tissue of the lungs over a greater or less extent. *Morgagni* has alluded to this sort of infiltration of air into the pulmonary tissue.

M. *Breschet* is of opinion that the frothy discharge from the mouth is not mere saliva (the salivary glands seldom exhibit any abnormal appearance), but is rather a diseased secretion from the fauces, pharynx, &c.—*L'Experience, No.* 171.

We observe that a well-marked case of hydrophobia was recently admitted into La Charité hospital under the care of M. *Bouillaud.* The disease came on nearly four months after the bite of a dog, the patient's health having remained quite good during the interval.

The appearances found on *dissection* were, as usual, very unsatisfactory. Nothing abnormal was to be seen in the mouth, fauces, or pharynx. Towards the lower part of the œsophagus there were several small ecchymosed spots on its mucous surface. All the abdominal viscera appeared quite sound. The lungs and heart also were sound. The meninges of the brain were somewhat congested; but the cerebral substance was unaltered. The medulla oblongata seemed to be a little softer than usual: the medulla spinalis and its membrane were sound.—*Gazette des Hôpitaux.*

---

### MAJENDIE'S METHOD OF TREATING NEURALGIA.

The remedy, *par excellence,* recommended by M. *Majendie* in the treatment of obstinate neuralgia of the face and other parts, is electro-acupuncture. The needles should be made of an unoxydisable metal, and therefore those of platina are to be preferred. With respect to the mode of introducing them, it is better to push them at once and with a sort of plunge, than to endeavour to drill them more slowly. In most cases two needles are quite sufficient; one near the origin of the nerve, and the other near its termination or expansion. The former is then to be connected with the positive wire, and the latter with the negative wire of a galvanic apparatus. The patients usually describe the sensations experienced as if a spark or stream of lightning passed instantaneously along all the divisions of the nerve: at the same time the muscles of the part are thrown into contractions. The application is not to be continued beyond a few seconds, except in some severe cases, in which a continued stream must be maintained for some time. M. *Majendie* gives the preference to *Clarke's* electro-magnetic machine, as being altogether more convenient than any other for the purposes of electro-acupuncture.

If the neuralgic pain leaves one branch of a nerve to fix itself upon another branch, or upon another nerve, one or both needles are to be withdrawn, and should be inserted along the course of the nerve newly-affected.

Several cases of supra-orbital neuralgia are adduced, in which the employment of electro-acupuncture speedily dispelled all suffering. The following one, in which the superior maxillary branch of the fifth pair was the affected nerve, may deserve to be noticed.

M. *Thelin* had been subject to frequent attacks of most severe neuralgia, affecting the superior maxillary nerve of the left side, when he first consulted M. *Majendie.* The pain in the gums, lips, cheek, and ala nasi were insupportable; the patient could scarcely utter a word, and, as for mastication, that was impossible. All methods of treatment had been tried, and all tried in vain. What with having many of his teeth extracted, and being leeched and blistered, and physicked for months and months at a time, his constitution had suffered severely. He consulted M. *Majendie* on the 5th of March, 1838: at one sitting of a few minutes the pain was *chassé.* Since that period, whenever the neuralgia returned, he repaired to M. *Majendie,* and always left him cured of his suffering. It is now several months since he has had an attack.

In the second volume of our author's lectures on the nervous system, he has related two cases of severe neuralgia affecting the tongue; in one of which the disease had lasted for four, and in the other, for one year...... "A very fine platina needle was inserted into the trunk of the facial nerve, where it enters the parotid gland, and another was inserted into the affected side of the tongue. In this manner I was sure to act on the seventh and fifth pairs of nerves, since I punctured the trunk of the first and the lingual branch of the second. The needles were then connected with the wires of *Clarke's* machine. In one of the patients the pain in the tongue immediately ceased, but it fixed itself on the mental branch of the inferior maxillary nerve. The needle was forthwith withdrawn from the tongue and inserted over the *foramen mentale.* The pain was driven from this point, but it was almost immediately transferred to the infra-orbital nerve. The needle was, therefore, introduced over the aperture from which it escapes. The enemy was thus pursued from one point to another, and ultimately was expelled before the patient left my house. In the other case, the pain, when driven from the tongue, took refuge in the sub-orbital nerve; driven from this, it returned to the tongue, whence it was again dislodged. Ultimately the patient was quite cured."

Certainly such practice is infinitely superior to that of attempting to divide each nerve, that becomes successively affected, as practised, for example, by M. *Roux* in a recent case, where he divided first the mental branch of the inferior maxillary, then the lingual, and, lastly, the sub-orbital nerve—the enemy, however, retreated to the ethmoidal, where the knife of the surgeon could not reach him.

"In such a case," says M. *Majendie,* "I pursue the pain, not with the bistoury, but with the galvanic current. Even should it fix itself upon the ethmoidal nerve, I should insert one needle into the nostril, and another into the orbit, along the upper part of its internal wall, at the place where the external nasal traverses it, thus attacking it both near its origin and its termination.—*Gazette Medicale.*

*Remark.*—We have no doubt that electro-acupuncture will relieve the suffering in many cases of neuralgia, which are unconnected with structural disease of any part; but it is more than probable that the relief will be only temporary, unless appropriate constitutional means are employed at the same time.—*Rev.*

---

### M. Biot on the Diagnosis of Diabetes Mellitus by the Optical Appearances of the Urine.

M. *Biot,* the distinguished natural philosopher, informs us that, Dr. *Mandl* having enquired of him, if by the observation of the rotatory power the presence of sugar could be detected in the urine, he has performed numerous experiments with this view on the urine of patients labouring under diabetes mellitus. Chemistry enables us to obtain from such urine a substance, which is fermentable, and whose characters of solubility, of fusibility, and of crystalline appearance, are in every respect quite identical with those of solidified sugar from the grape, and of that produced by the prolonged action of sulphuric acid on starch. Now this fermentable matter, so obtained, is sometimes sweet or saccharine to the taste, and at other times it is altogether insipid. Hence some authors, as M. *Bouchardat* in his excellent memoir in the Revue Medicale for June, 1839, have been inclined to conclude that there are two kinds of sugar in diabetic urine—one sapid, and the other insipid. It is, however, more probable that tastelessness of the fermentable extract is owing to the union of the saccharine substance with other ingredients of the urine, such as the lactate of urea, the chloride of sodium, and extractive matter: this opinion is confirmed by the results of optical examination. For the purification of the insipid product, by repeatedly washing it with alcohol, leaves as a residue a sapid sugar, which is quite analogous in appearance with the sugar of fecula, susceptible of fermentation like it, and which exercises a rotatory power of the same character.

"My first care," says M. *Biot*, "was to ascertain if healthy urine ever presented traces of rotatory power. The result of my observations has been that it does so never, or at least very rarely, and then accidentally and scarcely perceptibly. I long ago satisfied myself that urea, which enters so abundantly into the composition of healthy urine, exhibits no appreciable rotatory power. I had also found in diabetic sugar a rotatory power of the same kind (de meme sens) and of the same intensity as the sugar of starch—a fact which accords with the identity of ponderal composition, which chemistry attributes to them."

...... "I examined the urine of a diabetic patient, under the care of M. *Breschet*. He had been for some time subjected to a diet of animal food chiefly, and had derived so much benefit from it, that he was considered to be on the road to a recovery, although still affected with *diabetism*. His urine exhibited a power of considerable rotation, directed towards the right hand of the observer, consequently in the same direction as the solid sugar of diabetic urine obtained by evaporation. Its deviation, observed immediately with the naked eye, through a tube of $347^{mm}$, 6 in length, was $+ 10^{c}$, 6.*...... In a case under the care of M. *Rayer*, the rotatory power of the urine, observed in a tube of the same length, exhibited a deviation to the right hand of $18^{\circ}$, 5. This urine contained nearly twice as much saccharine matter as that of M. *Breschet's* patient. If this sugar might be considered as free, or as combined with substances which do not alter its rotatory power, the urine must have contained from 110 to 120 *grammes* in the *litre*—a proportion which does not exceed what M. *Bouchardat* has met with in extreme cases of the disease.

But to estimate in this way, with exactitude, the ponderable quantity of saccharine matter by the mere extent of the deviation observed through a liquid medium, it is necessary that the two conditions now specified should have been previously determined by the chemist. It is for this reason that, until such analytical examination has been made, I restrict myself at present to merely suggesting the optical diagnosis as a means of comparison."

M. *Biot* then mentions some other circumstances, which confirm the opinion that diabetic sugar is strictly identical with that obtained from fecula.

...... "M. *Rayer's* patient was seized with a pneumonia, while under treatment for the diabetes. The animal diet was, therefore, obliged to be suspended. The urine, under the influence of the inflammatory action, became of a deeper colour, and was found to contain much less sugar. Five days after the invasion of the inflammatory symptoms, and seven days after the suspension of animal food, the urine was found to exhibit no traces whatever of rotatory power: consequently all secretion of sugar had ceased.

* We give these figures as they stand in the French memoir, as we might commit an error in reducing them to an English standard.

How much more easy it would have been to have treated this case, had the poor fellow come to the hospital at the commencement of his disease, when the simple inspection of his urine might have proclaimed at once the danger of his situation!

In another case from the practice of M. *Rayer*, that of a child in whom there was a copious diuresis, accompanied with extreme thirst, &c. I examined the urine optically, but could not discover any sign of rotatory power. Now on evaporating it, we found that there was exceedingly little residue left, and that this was not fermentable. The optical and the chemical examination corresponded, therefore, in their results. It is, without doubt, easy in such a case to ascertain the nature of the urine by evaporating it and testing the residue; but it is still more easy to introduce some of the urine into a tube, and arrive at the same conclusion by simple inspection."

M. *Biot* suggests to physicians, that by following this plan, the optical examination of the animal fluids, they might ascertain whether the serum of the blood &c. in diabetes contains any free saccharine matter. He has already found that the serum of healthy fluid has a rotatory power directed to the left hand—in consequence of its containing albumen, which acts in this direction. If the serum contained any sugar, we may expect that its rotatory power should be considerably weakened, or perhaps altogether changed. A similar occurrence may be expected in the urine itself, when it has become albuminous. These are questions well deserving the immediate attention of the scientific physician.

M. *Biot* concludes his remarks with these words:—

"The optical characters of the urinary secretion will furnish an easy, sure, and exact means of diagnosis, to enable us to ascertain in a moment its diabetic condition. In this way, we may recognise the commencement of the disease from its earliest stage, detect at once its different peculiarities, and follow it through all its phases. It will then be easy to discover the immediate effects of regimen and diet, as well as of any medicinal agents that may be administered."—*Gazette Medicale.*

---

### Microscopic Observations on Milk.

M. *Donné* has recently communicated a memoir on this subject to the Royal Academy in Paris. A committee consisting of M. *Moreau*, professor of midwifery to the Faculty of Medicine, M. *Baron*, physician of the Foundling Hospital, and MM. *Orfila, Velpeau, Blandin* and *Louis*, was appointed to report upon it; and the following are the main conclusions of their report:—

1. The *colostrum* has microscopic characters which distinguish it from genuine milk: it may be recognised by *granity* (graniteux) corpuscules very distinct from the globules of milk, and by a peculiar arrangement of these last-named globules attributable to the presence of a mucous matter which connects them together.

2. In the normal state, and in healthy nurses, the milk does not exhibit any traces of colostrum, from the tenth to the twenty-fifth day after delivery.

3. We find the elements of the colostrum in the milk of certain nurses at much more distant periods after delivery; and this character constitutes one kind of alteration of the milk, which renders it unwholesome to the infant beyond a certain age.

4. The microscope enables us to appreciate the nutritive richness of the milk by the number of the globules, which are always in proportion with the other constituents of this fluid, (the caseum, sugar).

5. All the globules of the milk are formed of fatty matter, as is shewn by their solubility in ether; they are not composed, as it has hitherto been generally alleged, in part of butter and in part of caseum.

6. The milk of women, as that of the cow, &c., restores the blue colour of reddened litmus paper; it is therefore not acid, as stated in most works on chemistry.

7. The milk of good healthy nurses always exhibits numerous globules unadherent to each other, distinct, and without admixture of foreign bodies. Although there are some of all diameters from the 500th to the 50th part of a millemetre, the greater number are of a medium size.

8. Whenever milk does not exhibit these characters, and when its globules are confused, agglomerated together by a mucous matter, and do not float about freely, and without adherence to each other on a plate of glass, or when they are mixed with foreign bodies (granular bodies or mucous globules,) we must regard it as more or less abnormal and unhealthy. We observe it so in nurses who have an engorgement or any local disease of the mammæ, and in those whose general health is disturbed.

9. The presence of pus and of blood in milk is readily determined by microscopic examination, as well by the difference in aspect and organisation of the globules which enter into the constitution of these three liquids, as by the different effects produced upon them by ammonia and æther. The globules of milk resist the action of ammonia, while they are entirely soluble in æther: it is the very reverse with the globules of pus and of blood.—*Gazette Medicale.*

*Addend.*—We observe, in a recent number of the same journal, a note on the influence of different articles of food on the milk of cows and goats. Turnips, it is said, render the milk lighter and of more easy digestion than the common fodder, while beet-root makes it extremely rich and substantial. The convalescence of the *Count of Paris,* the infant grandson of *Louis Philippe,* is attributed to the milk of a cow fed on turnips having been substituted for that of his nurse, the latter having been found to be not sufficiently nutritious. "The success was immediate and complete, the young prince being restored to perfect health." —*(Rev.)*

---

### DIEFFENBACH ON THE CURE OF STAMMERING BY OPERATION, WITH REMARKS.

The distinguished surgeon of Berlin has addressed a lengthened letter to the Institute of France on the subject of curing impediments of speech by means of a surgical operation. We shall give an abstract of its contents before offering any remarks of our own.

M. *Dieffenbach* tells us that, after long consideration of the subject, the idea quite suddenly (tout à coup) came into his mind that "the change produced in the innervation of the muscles of the tongue, by dividing the muscular part of this organ, might probably effect a cure of stammering." What suggested the idea to him was the circumstance of a person who squinted with both eyes, and who happened to be also affected with stammering, coming to his consultation to have an operation performed on her eyes. He has subsequently observed that these two defects are often co-existent in the same person.

"As I believed that the cause of stammering was dynamic, and consisted in a spasmodic state of the air-passages, and more especially of the glottis, communicating itself to the muscles of the tongue, and of the face and neck, I inferred that, by interrupting the innervation to the muscles involved in this abnormal condition, we might succeed in modifying, or even completely suspending it. The obvious way of doing this, was to make a transverse section of the root of the tongue."

He has tried three different modes of operating :

1. The transverse horizontal section of the root of the tongue.

2. The transverse subcutaneous section of the root of the tongue, with the conservation of the mucous membrane.

3. The horizontal section of the root of the tongue, with excision of a triangular piece of its substance.

"I had anticipated much greater benefit from the last than from the other two methods of operating, because it at once produced a shortening of the tongue, and enabled the patient to move it up easily against the roof of his mouth—a movement which is always strongly inculcated by all those who profess to cure stammering. As, however, it was a more serious operation than the other two, I wished to give a fair trial to them also. The instruments required for these operations are several kinds of forceps, a hook provided with a handle, a long narrow curved bistoury, and several curved needles armed with ligatures."

The first operation performed by *Dieffenbach* was on the 26th Jan. of the present year. He adopted the third plan, that of excising a triangular portion of the root of the tongue. The patient, a boy 13 years of age, being seated on a chair and his head resting on the breast of an assistant, the tip of the tongue was laid hold of and drawn out with *Muzeux's* forceps, which were held by one assistant, while another with a blunt hook kept back the angle of the mouth. The operator then introduced the point of the knife, with its cutting edge directed upwards, into the left side or margin of the root of the tongue, and, having pushed it on through its substance to the point opposite to that where he had entered it, he then made a complete section from below upwards. Having then fixed the posterior edge of the wound with a strong suture, he seized with a forceps, provided with points, its anterior edge, and having thus compressed it laterally, he cut away a triangular piece of the entire thickness of the tongue. This done, he drew the posterior edge as far forward as he could, and by means of six strong points of suture he attached it to the anterior edge, carrying each thread deep into the substance of the tongue, so as to guard against subsequent hæmorrhage, which had been very considerable during the operation. We are told that, immediately afterwards, the boy was able to pronounce several words without at all stammering, (!) although the contortions of the face still continued. By the seventh day, the wound was completely healed: "there was not the slightest stammering, nor any convulsive movement of the face or lips during speaking: the pronunciation was altogether pure, sonorous, and rapid; and, even with strangers or when surprised, there was no impediment or interruption. Even the most difficult words he could pronounce as quickly as another person. Baron *Humboldt* examined him on the tenth day after the operation, and satisfied himself of the perfectness of the cure."

The *second* case was treated in the same manner as the preceding one, and with similar success. The youth, 16 years old, had stammered from the sixth year of his age, after an attack of pneumonia : most of his brothers and sisters were affected with the same impediment. Eight days after the operation, not only was the wound perfectly healed, but the tendency to stammering was entirely removed.

Equal success attended the performance of this operation in twelve other cases !

M. *Dieffenbach* has performed the operation of—

*Simply dividing horizontally the Root of the Tongue* in four cases; in one it failed,* and in the other three the incisions had not quite healed at the date of

---

* It is worthy of notice that in this case, in which *Dieffenbach* himself admits that the operation failed, the stammering entirely vanished immediately after the operation: it returned however, as bad as ever, in the course of two or three days. Is not this remark applicable to not a few of the cases which have been trumpeted forth in this country as cures?—*Rev.*

his report, although the stammering had already entirely ceased in all of them. The operation consists in first securing the tongue, then making a transverse incision across its basis, and lastly bringing the edges of the wound together by several stitches.

The *third* operation, proposed by *Dieffenbach,* consists in

*The subcutaneous transverse Section of the Root of the Tongue,* leaving its mucous membrane undivided. He reasoned thus on the advantages and disadvantages of this mode of operating, before proceeding to adopt it in a particular case, where the tongue seemed to be shorter than usual: "On the one hand it seemed to me advantageous, in so far as that the posterior part of the tongue could not be well drawn forward, which necessarily rendered the application of any sutures more difficult; on the other hand, it seemed to me doubtful, in consequence of the difficulty of arresting the hæmorrhage, since it was scarcely possible to employ compression, and there was, therefore, the risk of a quantity of blood accumulating in the subcutaneous (or rather submucous) wound of the muscles of the tongue. Lastly, it appeared to me less certain of success, because there was not necessarily any shortening of the tongue produced by it, a condition which I deemed indispensable to effect a cure of stammering. The success of my first trial has, however, greatly exceeded by expectations."

The operation is entirely the same as the preceding one, with the exception of the mucous membrane of the tongue being not divided: the narrow falciform bistoury is plunged into the substance of the tongue at one side and carried across to the opposite side, dividing in its *trajet* the entire thickness of the muscles; it is then withdrawn, without dividing the mucous membrane, as in the subcutaneous operations practised so extensively by M. *Guérin.*

"The blood streamed out from both lateral wounds, as if it came from a large arterial trunk, and the tongue became much swollen by the blood which accumulated in the wound. To contract the width of this, I passed a strong suture from behind forwards, through the substance of the tongue, and thus closed the two lateral wounds or punctures made by the bistoury. The deglutition was considerably impeded for several days afterwards. The suture was removed on the fourth day, the union being complete through the entire depth of the wound, and on the eighth day the patient left his room. He no longer stammers in his speech, except with certain words difficult of pronunciation. This mode of operating," adds M. D., "presents more difficulty than the other two."

M. *Dieffenbach* closes his memoir with some practical remarks on wounds of the tongue in general. He observes that they almost always exhibit a marked tendency to unite very rapidly, provided their edges be kept together by means of sutures. To effect this, the sutures must be strong, sufficiently numerous, and inserted deep, so that no empty un-united space exists at the bottom of the wound. Although the wound is usually united by the third or fourth day, it is well not to remove the stitches for a day or two longer.

The chief danger of wounds of the tongue is the risk of serious hæmorrhage; this is another reason for the employment of strong deep sutures, at short distances from each other.

M. D. tells us that he has tried, in several instances, the operation of dividing the frænum linguæ for the relief of stammering; but that it is entirely useless. His concluding remarks are so amusingly caustic, and withal so very just, that we must not deprive our readers of the pleasure of perusing them.

"At a time when it is so fashionable to modify every operation, however simple and good it is acknowledged to be, surgeons will have an ample field to propose modifications of the three methods which I have described above, and, also, to invent new instruments. They will, no doubt, be making incisions crucially, transversely, above, and below the tongue; they will be using caustics of various sorts; they will be employing curved bistouries and scissors of a new fashion, and, also, other hooks, and other kinds of forceps; even to the very handles of the instruments, there will be some change or another introduced, for the purpose of permitting the light to fall better upon the cavity of the mouth. This new operation furnishes to the surgical antiquarian an occasion to devise new names; to them I leave the care of baptising it with a Greek appellation."—*Gazette Medicale*, 13 Mars, 1841.

*Remarks.*—It does not, we think, require the eye of a prophet to foresee that the operation—for the three methods proposed are only modifications of one—recommended by *Dieffenbach*, can scarcely ever be adopted by the scientific surgeon for the cure of stammering. Independently of the pain and difficulty attending its performance, and the risk of the serious hæmorrhage which may be induced, it is surely based on a merely conjectural, if not upon an erroneous, foundation. For, what reason is there to imagine that stammering depends on a shortening of the tongue? If such were its cause, how are we to account for the speedy cures which are every day effected by mere instruction, without any surgical operation being performed? And how shall we explain the immediate effects of surprise in aggravating the infirmity and of quietude in allaying it? That stammering is in most cases a purely spasmodic complaint, arising from some want in the normal equilibrium and co-operation of the muscles of speech, cannot, we think, for a moment be doubted; and that its cure is to be based upon the proper vocalisation of the voice, seems to be established by the circumstance of stammerers being usually able to pronounce the words in singing with perfect ease, and without any impediment. What then should induce us to fly to a surgical operation, and to the cutting and carving away of the tongue, or the tonsils and the uvula, or the frænum and base of the tongue, to relieve a defect which is certainly purely functional? And here it may be necessary to caution our readers not to allow their minds to be too much influenced by the reports of *immediate* success after the operations either of *Dieffenbach*, or of MM. *Amussat* and *Lucas*, whose plan consists in dividing the frænum linguæ and the hyo-glossi muscles, or of Mr. *Yearsley*, who has been very busy excising the uvula and portions of the tonsils of every stammerer that comes within his reach.

That any operation or plan of treatment, which causes a certain amount of pain, and will therefore have the effect of throwing the muscles of the throat and mouth into new contractions, may be followed by some relief to the stammering, we are not at all surprised: but we much doubt that the relief will almost ever be permanent.* As well might we propose to remove the convulsive movements of chorea, or the paralytic affections of hysteria, by surgical operations on the affected members; the cases appear to us nearly parallel.

In conclusion, we venture to predict that ere another quarter or two have passed, we shall hear very little of curing stammering either by cutting the tongue either above or below, or by excision of the uvula and tonsils.—*Rev.*

---

### Fatal Wound of the Vertebral Artery.

One of the porters of the prison at Limoges was stabbed on the right side of the neck, at about an inch below, and a little in front of, the mastoid process. The

* The labours of the medical critic would be much lessened, if the foolish practice, more common we confess on the continent than in this country, of reporting cases of surgical operations almost immediately after their performance, and often before their ultimate results can be known, was discontinued: it is only worthy of an advertising quack.

assassin had struck him from behind, and the direction of the wound appeared to be transversely from without inwards. The hæmorrhage was frightful, and was evidently arterial. As the temporal and facial arteries on the wounded side still continued to pulsate, it was inferred that it was not the external carotid that had been divided. Was it the vertebral? or was it the internal carotid?

M. *Voisin,* deeming that it was the latter vessel, proposed to tie the common carotid, and immediately performed the operation. But the hæmorrhage from the wound continued as profuse as ever, and could only be checked by firm compression somewhat above the wound. At length it ceased; and on the sixth day after the accident, the wound was almost completely cicatrised. Three days subsequently, after walking about for a minute or two, a slight hæmorrhage returned; but this was quickly arrested by repeating the compression as before. On the following week, however, again it broke out; and, on removing the apparatus, the skin was found to be somewhat gangrenous, where the firm pressure had been made. Although the bleeding did not return, M. *Voisin* proposed to perform a second operation with the view of securing the upper end of the vessel that had been wounded. On making an incision through the integuments, a frightful stream of blood poured forth. This could not be stopped by compression either above or below the seat of the wound; but it was checked by keeping up firm pressure along the course of the occipital artery, behind the mastoid process. M. *Voisin,* supposing therefore that it was this vessel that had been injured, immediately set himself to tie it at its origin. The sterno-mastoid muscle was therefore divided at its upper insertion and the digastric was exposed, when the hæmorrhage returned with redoubled violence, and could be only arrested by pressure with the fingers at the bottom of the wound. As it was utterly hopeless to attempt placing a ligature, all that was done was to fill the wound with a compress and secure it in its place with a bandage. From this time to the period of his death, which occurred two days afterwards, the patient complained of pain and sense of powerlessness in his right arm.

*Dissection*....... "The vertebral artery was found to have been almost completely divided at the point of its exit from the third cervical vertebra, where it forms an arch to reach the aperture in the transverse process of the second vertebra."......

*Remarks.*—The error in diagnosis of M. *Voisin,* as to the vessel that was wounded in the preceding case, is quite excusable; but certainly we must confess that in our opinion the second operation was quite unnecessary, and decidedly hastened the fatal event. To attempt ever to tie the vertebral artery must surely be unnecessary; the only thing that can be done under such circumstances, is to trust to compression, maintained rather by the hand of assistants than by any bandages applied round the neck and head.—*Gazette Medicale.*

---

### ON THE TREATMENT OF RECTO-VAGINAL FISTULÆ.

M. *Petrequin,* the chief surgeon of the Hôtel Dieu at Lyons, whose name is not unknown to our readers, has published at great length a case of that most distressing and often intractable accident, rupture of the recto-vaginal septum, successfully treated by operation. He justly remarks, that it is to M. *Roux* that surgery is indebted for the most valuable suggestions and precepts on this subject. It was he who first substituted the *quilled* for the *interrupted* and the twisted sutures in the approximation of the edges of the wound, and it is to this important modification that the success of his operations is mainly attributable. Two years ago he had performed and adopted this practice in eleven cases; in six of these the patients were cured by the first operation; in two it required to be repeated a second time; two patients died; and in the remaining case the wound gave way on the 14th day after the operation, during the efforts of defecation. No other mode of operating presents so satisfactory a result—8 cures in 11 cases.

The following is an abridged report of M. *Petrequin's* case.

A woman, 28 years of age, had a protracted and severe labour with her first child; when the child was expelled, the perineum and recto-vaginal septum were found to be lacerated. Eight months after this date, she was admitted into the Hôtel Dieu at Lyons, under the care of M. *Petrequin.* The entire extent of the perineum was lacerated, and the laceration extended into the rectum for 14 or 15 lines in depth; the constrictor muscle of the vagina and the two sphincters of the anus, with the exception of a few fibres of the upper one, were divided; the double ano-vulvar orifice was confounded in one large opening, and constituted a veritable cloaca, into which the excretions of the alimentary, the urinary, and the genital organs were received. The vagina and the os tincæ were the seat of minute ulcerations; and the general health had suffered considerably. The patient was therefore treated for some time with injections of a weak solution of the chloride of lime; and the os tincæ was occasionally touched with alum. After some time the general as well as the local health became much improved, and M. P. proceeded to the operation. The edges of the fissure being very finely pared, partly with the bistoury and partly with scissors, he inserted three curved needles, each armed with a double stout thread, through the two sides of the wound, taking good care that they penetrated to its deepest part. The ends of the ligatures were subsequently secured over two pieces of caoutchouc bougie placed along each side of the fissure.

As the lips of a wound always gape somewhat when a *quilled* suture is used, M. P. took the precaution of passing two stitches of the *interrupted* suture, so as to bring them in closer approximation.

The bowels having been kept well open for several days before the operation, opium, in the form of extract and syrup, was given afterwards to confine them, and the patient was put upon a very low regimen in order to prevent the necessity of defecation for several days. A catheter was also left in the bladder, so that the patient could relieve herself, when necessary, by merely withdrawing the plug. The vagina was freed from any purulent or other discharge, by means of mild emollient injections, morning and evening. On the third day, the patient had a liquid stool without any prejudice to the sutures. There was no evacuation of the bowels again until the eighth day, and then without much effort: the edges of the cicatrix at the anus were slightly disunited in consequence. On the following day the stitches and the pieces of bougie were removed, the cicatrisation of the anterior three-fourths of the wound, at least, being by this time nearly complete: the patient began to take vermicelli soup, and the dose of the syrup of poppies was reduced to 16 *grammes.* On the eleventh day, there were two stools; and again there was a very slight laceration of the cicatrix near the anus; but the union in the perineum seemed to be complete.* On the eighteenth day, a superficial ulceration made its appearance in the perineum: by touching it however with a portion of alum it disappeared in the course of two or three days.

As remarked by M. *Roux*, there remained in all his cases a small aperture of communication between the rectum and the vagina for a considerable time after

---

* M. *Roux* has remarked on the tediousness of the healing of the fissure near to the anus:—"In all the cases, the edges of the division next the anus were disunited or rather separated, although ultimately they closed. At this point the wound was slightly gaping, and looked like that which is caused by the operation for fistula ani. But this fissure has in every case eventually disappeared, and the anus has recovered its normal condition."

the perineum had completely united. M. *Petrequin* had for some days hoped that he had been more fortunate, and that the recto-vaginal septum had perfectly cicatrised, as no portion of a fluid injected into the vagina passed into the rectum. But this hope proved in a few days to be deceptive. With the exception of this minute aperture between the two passages, the whole extent of the fissure was completely cicatrised, when the patient left the hospital on the 15th of August, 35 days after the operation. Unfortunately the poor creature caught cold when she returned home, and was seized with fever; she was brought back to the hospital, a fortnight after her leaving it, in a dying state. On dissection, it was found that the circumference of the anus had been attacked with ulceration, which had been extending deeper and deeper in, so as to affect the sphincter muscle and give rise to two small fistulæ. The perineum however remained firm and perfectly united; and the recto-vaginal septum was closed, with the exception of one point, where an oval aperture, which communicated with the two passages, existed: its edges were hard and resisting. Thus the large lacerated opening, which originally existed, had been reduced to a small fistulous aperture; and we have every reason to believe, from the experience of M. *Roux*, that this would have gradually closed.

In closing the report of this case, it is impossible not to admit the great superiority of the *quilled* over every other kind of suture in the treatment of recto-vaginal fistulæ. The introduction of this great surgical improvement we owe entirely to the sagacity of M. *Roux*.—*Gazette Medicale.*

---

### RESULTS OF 643 CASES OF LITHOTOMY IN NAPLES.

These 643 cases occurred during the eight years from 1821 to 1828, and during the year 1839. Of the entire number 625 were in males, and 18 in females; 321 were in children, 262 in adults, and 60 in old people. (The exact periods of life are not stated.) The operation, which seems to have been the lateral one on all occasions, proved successful in 541 cases, and was followed by fatal consequences in 100 cases. This is very nearly the average rate of success of lithotomy in the practice of the most distinguished operators of recent times.—*Gazette Medicale, No.* 52.

---

### TREATMENT OF CIRCOCELE BY OPERATION.

M. *Jules Roux*, one of the naval surgeons of the arsenal at Toulon, informs us that he has succeeded in effecting a cure in several cases of this unmanageable complaint by adopting the operation recommended by M. *Reynaud*, the *chef* of the surgical staff there, to effect the obliteration of the varicose vessels. The report of the following case will enable our readers to understand the nature of this operation.

"A sailor on board the Montebello ship of the line, aged 27 years, applied to me for relief from a large circocele, which had long given him great inconvenience. He stated that from his infancy he had been affected with a varicose dilatation, not only of the spermatic veins on the left side, but also of the veins of the left leg, extending upwards along the thigh to the groin.

The spermatic veins form around the testicle, which is seemingly quite healthy, numerous nodose plexuses, of which the *ensemble* forms a tumor as large as the closed fist. The vas deferens is easily distinguished and separated from the venous mass which adjoins it. When the patient is at rest, and the atmosphere is dry, the tumor diminishes considerably, and gives him but little annoyance; but when he stands up or moves about, especially if the weather be damp and relaxing, the veins become much enlarged, and the scrotum and cord are elongated."

The following operation M. *Roux* performed on the 15th of August.

Separating the vas deferens from the bunch of varicose veins, and keeping it apart with the thumb and forefinger of his left hand, he took a large curved needle armed with a double waxed ligature, and inserted it through the integuments of the scrotum, carrying it round the cord, and making it come out at the opposite point, but leaving the threads in. A circular compress of lint was placed between the threads and the skin of the cord, so that the compression which was made by tying them was more even and uniform. The pain was considerable; but it gradually subsided within the course of an hour, and only returned when the patient coughed or sneezed. Next day, the ligature was undone, and re-tied rather more firmly; this was repeated on the 17th, 20th, 22nd, 24th, 26th, 28th, and 29th of August, and again on the 1st of September, as the soft parts gave way under the constriction of the thread and cicatrized over it. Each time it caused considerable pain, which lasted from 10 to 30 minutes. On the third day after the operation, the edges of the little wounds became inflamed, and the inflammation gradually affected the entire scrotum and also the testicle; but no constitutional fever was induced. Emollient laudanised poultices were applied. On the second of September, the nineteenth day after the operation, the soft parts within the ligature being nearly divided, the narrow pedicle was cut across with a narrow bistoury introduced along the course of the ligature. Six days subsequently the patient left the hospital quite cured, and resumed his duties on board ship.

Two months afterwards he was examined. The testicle was of its ordinary dimensions and consistence; below the whitish linear cicatrix, which looked like a circular furrow, five or six cords, smaller and softer than the vas deferens, were distinctly felt: these were the impermeable spermatic veins. (Query, how is the testicle nourished, if the spermatic cord, with the exception only of the vas deferens, be divided across with a ligature, as in the preceding operation?)

M. *Roux*, it seems, has performed the same operation with perfect success in several other cases of circocele: they are recorded in two memoirs, one in the Gazette Medicale for 1837, No. 52, and the other in the Journal des Connoissances Medicales for February 1839. He deems it altogether preferable to the operations recommended by M. M. *Velpeau* and *Breschet:* the merit of it belongs to his *chef*, M. *Reynaud*, who in his opinion has "perfectionné ce point de pathologie chirurgicale."—*Gazette Medicale.*

---

### Results of Amputations in the French Army at Algiers.

*Statistical Report of the Amputations performed in the Hospitals as well as in the Camps, during the Years* 1837-8 and 9.*

These amputations amounted to 63; viz.

| | |
|---|---|
| At the shoulder-joint | 6 |
| At the elbow | 2 |
| At the wrist | 6 |
| At the knee | 1 |
| Of the foot, partial | 1 |

* The operations performed in the campaign to Constantine in 1837 are not included in this report. The minor amputations also of fingers and toes are not given.

| | |
|---|---|
| At the metatarsal joint - - - | 1 |
| Of the thigh - - - - - - | 16 |
| Of the leg - - - - - - | 7 |
| Of the arm - - - - - - | 15 |
| Of the forearm - - - - - - | 8 |

Of these 63 cases, 17 proved fatal; thus exhibiting a mortality of one in between three and four of all. It must however be mentioned, that of the 17 fatal cases, three of the patients were operated on under circumstances which held out very little hope of recovery; one received a fracture of the cranium as well as the injury of the limb which required amputation, one died of hæmorrhage during his transport from Tunis to the coast, and one died from the effects of an overfeed when he was going on most satisfactorily—so that the mortality attributable to the operation cannot perhaps be estimated higher than at 11 or 12 out of the 63 cases.*

(During the campaign of Constantine, when the army was exposed to the greatest hardships and to the most trying severity of weather, the mortality after amputations was on some occasions appallingly high. Thus of ten cases operated upon at Medeah in July 1837, one only proved successful; and of 62 at Blidah 39 proved fatal. The mortality was in proportion to the magnitude of the operation: thus of 30 amputations of the thigh, four only proved successful.)

Of the 63 cases, the operation was performed primarily or immediately after the injury in 44, and secondarily or consecutively in 19. Among the former, there were 32 cures and 12 deaths; and among the latter there were 14 cures and 5 deaths. It would therefore seem that quite as great success attended secondary as primary amputations. This however does not at all contradict that precept of our best military surgeons, at the head of whom we may justly place Baron *Larrey*, that immediate amputation should generally be performed after severe gun-shot wounds of the extremities: much anxiety and great inconvenience and trouble will thereby be spared to the operator. Dr. *Guyon*, the author of these remarks, after citing a number of instances in which success attended his secondary or consecutive amputations, says:—" In my opinion, the secondary operation presents at least as many chances of success as the primary, if performed on parts more or less distant from the trunk, and many more chances if performed on parts nearer the trunk of the body, such as the thigh or the hip-joint. We know that as yet there is not a single successful instance of primary amputation at this joint. In the case recorded by *Guthrie*, the operation was not performed till 15 days after the receipt of the wound. A certain degree of constitutional depression is very obviously a favorable condition for the performance of so formidable an operation as removal of the thigh at the hip-joint. It has been performed, but unsuccessfully, three times in the French army at Algiers."—*Gazette Medicale.* 1841 T. IX, p. 103

---

TREATMENT OF ANCHYLOSIS BY RAPID EXTENSION.

Our readers are probably aware that a surgeon of the name of *Louvrier* was trying, in some of the hospitals in Paris, the practice of rapid extension of anchylosed joints by means of a powerful mechanical apparatus. We condemned the practice at once, as utterly inconsistent with all the principles of rational sur-

---

* Even at this estimate it would seem that the average mortality after amputations, at least in the military practice of the French surgeons in Africa, is nearly the same as that after lithotomy, as stated in the preceding article.

gery, and as attended with the most disastrous results to the unhappy victims who were experimented on.

As usual, the subject was communicated to the Royal Academy of Medicine, and became the theme of discussion among its learned members. An official report from the commission appointed to examine the practice has recently been published. The following *resumé* will enable our readers to appreciate its contents.

M. *Berard* (the reporter) said, that the operation of forcible extension of anchylosed joints had been performed in 22 cases: in three only had any troublesome consequences followed; in all the other cases the operation had no dangerous consequences (resultat facheux.) The greater number of the patients experienced, at the time of the extension, excessive sufferings; and in not a single case had the affected joint recovered a perfect freedom of movement. In some cases, a certain degree of luxation of the tibia on the posterior face of the lower end of the femur was remarked: this was partly owing to the resistance opposed by the patella, which had become agglutinated to the front of the condyles, and to the atrophy of the articular extremity of the tibia. As to the three cases in which the operation produced dangerous or fatal results, we are given the following particulars.

In the first unsuccessful case, which was the twelfth on the list, the anchylosis of the knee was complete, and the flexion of the joint so great that the heel nearly touched the buttock. The application of the extending apparatus was followed by a considerable laceration of the integuments, and a luxation of the leg on the posterior part of the femur, and subsequently by a profuse discharge of purulent matter which proved fatal three weeks after the operation. On dissection, the knee-joint was found full of pus; the anterior crucial ligaments were in a softened state; one of the posterior ligaments was torn across, and to its torn surface a portion of bone which had been detached from one of the condyles still adhered: several of the muscles also were lacerated.

In the second case, the patient experienced dreadful suffering at the time of the operation, and remained in a state of insensibility for some time afterwards. On the following day, an appearance of gangrene, attributable most probably to a rupture of the popliteal artery, shewed itself. Fortunately the gangrene became limited; and ultimately the patient recovered.

The third case occurred in a young girl, whose anchylosed limb was bent to nearly a right angle. The *redressement* being not complete, M. *Louvrier* applied a strong splint to the front of the joint, by means of which he proposed to complete the extension and prevent the shortening of the limb. On the following day however an eschar formed on the front of the knee, and the femur was found to have sustained a comminuted fracture: the patient died six weeks afterwards.

In another case, in which however the *redressement* was not followed by any accident, the patient died of a disease quite independent of the joint affection; and it was then found, on dissection, that the articular surface of the tibia was luxated on the posterior part of the femur, and also that the inner condyle had been fractured.

Considering therefore, says M. *Berard,* the comparatively small number of accidents after an operation apparently so frightful as the forcible extension of an anchylosed joint, the judgment which we should pass on the practice of M. *Louvrier* would not be so unfavourable, if its dangers were balanced by real advantages. But unfortunately it is not so; the limb remains almost immoveable, and has very little more freedom than one made of wood. On the whole we are inclined to draw the following conclusions.

1. That the application of M. *Louvrier's* apparatus is usually followed by an instantaneous rectification (redressement) of the anchylosed limb;

2. That this rectification is not ordinarily followed by any dangerous accident, either immediate or consecutive;

3. That the accidents however, when they do occur, are almost always frightful and generally prove fatal;

4. That none of the patients treated by forcible extension has recovered entirely a freedom of movement in the anchylosed joint.

" We should therefore reply to the minister that the apparatus of M. *Louvrier*, however ingenious it may be, is of extremely dangerous application, for it will be always impossible to determine beforehand the nature of the existing anchylosis or to foresee the conditions which might offer some chances of success in employing it."

M. *Larrey* expressed his concurrence in the conclusions of the report.

M. *Gerdy* condemned the apparatus of M. *Louvrier* altogether, and denominated his practice as worthy of a day-labourer or workman, rather than of a surgeon. The operation was violent, coarse, and unguided by any safe rules. " As a general maxim, we should never perform, on a human being, any operation, the consequences of which are more serious than the disease itself. In the present day surgeons are too apt to forget or despise this fundamental principle of surgery as well as of humanity; and too often we hear of rash experiments being performed in our hospitals, and in the presence too of students who have come to the metropolis to be educated, and who will hereafter carry into the provinces many a deplorable practice."

. . . . " I repeat," said M. *Gerdy*, " the surgery of the present times sins most by an excess of rashness; it resorts to operations at a mere venture, and without their being based on any sound and established principles; it operates everywhere and on all occasions. In this manner we may no doubt succeed in making a trade of our operations, (à faire de l'industrie, des operations mercantiles); but most assuredly we shall make no progress in our art, and science will inevitably be lost sight of in mere random speculation."

M. *Velpeau* adopted the conclusions of the report. He regarded the application of the proposed apparatus as highly dangerous, but nevertheless he did not entertain the same fears as most surgeons of the sudden rectification of the joint in anchylosis. The practice is not so dangerous as is usually imagined: we only want a *convenable* method of effecting it. He acknowledged that he had given M. *Louvrier* opportunities of trying his practice among his patients, and he did not at all regret that he had done so, as there was no other mode of ascertaining the results of new contrivances but by experiment.

---

### THE PROGRESS OF MEDICINE IN FRANCE DURING 1840, (continued.)

In our last number, (p. 530,) we gave the commencement of an abridged report of several papers by M. *Guerin*, the editor of the Gazette Medicale, on the progress of medical and surgical knowledge in France during the preceding year. We stated that they present but a superficial view of so interesting a subject; but that, however imperfect they are, they bring together a number of facts and statements, which may enable the English reader to form some idea of what is going on in continental medicine. We proceed therefore in our selections.

. . . . . . . .

Pathological physiology, in the department of auscultation, has received some interesting contributions from MM. *Piorry*, *Beau*, and *Raciborski*. The first of these gentlemen has successfully applied plessimetry to determine the dimensions of the aorta, and to complete the diagnosis, hitherto imperfect, in numerous cases of dilatation of this vessel. M. *Beau* has contributed several papers in support of his peculiar views, that the various respiratory sounds of health, and also their modifications in disease, are merely the resonances or echos of the sounds generated in the glottis backwards along the bronchial tree. Dr. *Raci-*

*borski* has endeavoured to simplify the history of the moist *rales*, of which there are so many subdivisions described by *Laennec* and other writers. After shewing how much the characters of a moist *rale* are influenced by the age, the condition of the patient at the moment of examination, &c. he proposes to recognise only one kind or species of it, the *bullar rale*, as the primitive or essential form. There is nothing analogous to the characters of this sound in the state of health; hence, whenever it is heard, we may be assured that a morbid condition is present in some part of the respiratory apparatus. Its numerous varieties depend upon the larger or smallei size of the bullæ, on the more or less viscid state of the mucous secretion, on the consequent facility of expectoration, &c.

. . . . . Pathogeny has been indebted to M. *Langenbeck* for a series of experiments which tend to prove the transmissibility of cancer from the human being to the lower animals. When cancerous matter had been injected into the veins of dogs, cancerous tubercles were found, on dissection some time afterwards, developed in the lungs. MM. *Rayer*, *Leblanc* and *Berard* have on several occasions repeated the inoculation of animals with glanderous matter taken from the human subject.

M. *Triberti* has pointed out the influence of light on the spasmodic phenomena of hydrophobia; it would seem, according to his statements, that hydrophobic patients can sometimes swallow in the dark with comparative facility. M. *Dezeimeris* has published a luminous memoir on the diseases of the epiglottis—and M. *Barthez*, *Roger*, &c. have shewn, from an extensive series of observations, that typhoid fever, hitherto regarded as of rare occurrence in children, is quite as frequent at this age as among adults. We must not omit to allude to the series of most valuable researches of MM. *Andral* and *Gavarret* on the changes of the blood in many of the most frequent diseases to which the body is liable.

. . . . . In surgical pathology, the labours of M. *Guerin*, in illustrating the hitherto obscure subject of deformities, deserve especial notice. He has shewn that they are almost all attributable to a spasmodic retraction, which has in course of time become permanent, of certain muscles or sets of muscles, and that by the subcutaneous division of the retracted muscles—an operation at once simple and entirely void of danger—the deformity may be generally removed. In the extraordinary case, in which M. *Guerin* divided no fewer than 42 muscles, tendons, and ligaments in the same patient at one sitting for the cure of various existing deformities, the operation was followed by no unpleasant symptoms, and by a very marked *redressement* of every part that was contracted.

M. *Bonnet* has drawn the attention of surgeons, in an especial manner, to the positions of the limbs in cases of diseased joints: he has shewn that, by injecting fluids into the articular cavities in the dead subject, we cause the limbs to assume those directions in which we had ascertained anatomically and experimentally that there was an increase or enlargement of the intra-articular spaces. From this fact he infers that the positions of the limbs, in diseases of the joints, are the result of effusions, which render such positions necessary to lodge the excess of the effused fluid. M. *Nichet* has continued his beautiful researches on diseases of the spine: he shews that the destruction of the inter-vertebral cartilages often commences in the centre of these disks, without however exhibiting any traces of tuberculous degeneration, and he assimilates the gradual destruction of these cartilages to that of the cartilages of diarthrodial joints. M. *Malgaigne* has examined every question connected with the important subject of herniæ at great length and with much ability; and M. *Velpeau* has pointed out a new variety of inguinal hernia, in which the protruded gut has forced a passage through the abdominal wall between the tendon of the rectus muscle and the umbilical artery, and thence penetrated into the inguinal canal, leaving intact the external ring, and raising the aponeurosis of the great oblique muscle. This surgeon has also been trying some experiments with the view of effecting a radical cure of reducible herniæ. His plan consists in scarifying the inner surface of the herniary sac, according to the *subcutaneous* method proposed, and so extensively adopted, by M. *Guerin* in performing the section of contracted muscles. M. *Velpeau*, it would seem, has not obtained complete success; but a M. *Sotteau* has been fortunate in one or two instances in which he followed a similar plan. And M. *Maisonneuve* has published an excellent paper on fractures of the fibula, and has proved that there are several other varieties of it besides those which *Dupuytren* and most surgeons have admitted. . . . . . .

Need we make any allusion to the new operation for the cure of squinting? Every journal, French and foreign, has teemed with suggestions and reports of cases. The operation is certainly an important addition to surgery; but there needs a good deal of discrimination in the selection of the cases in which it should be performed. Several surgeons have adopted the suggestion of M. *Guerin* to divide, according to the subcutaneous method, one or more of the muscles around old dislocated joints, in order to facilitate their reduction; and success in several instances has been thus obtained.

MM. *Dieffenbach* and *Veinhold* have reported two cases in which they effected the reduction of an old dislocation of the humerus by dividing (subcutaneously) the great pectoral muscle. M. *Guerin* has told us that he has made the subcutaneous section of almost every muscle of the body; and that, as already stated, in one patient alone he divided no fewer than 42 muscles, tendons, or ligaments at one operation, for various kinds of deformity, with the most satisfactory results.

### *Medical Pathology and Therapeutics.*

M. *Nonat* has published some interesting observations on the relation that exists between intermittent fever and enlargement of the spleen—a relation to which M. *Piorry* had previously called the attention of physicians—and has pointed out a useful indication for the exhibition of quinine in such cases. He suggests that the amount and frequency of the doses should be proportioned to the degree and duration of the splenic enlargement, and that the use of the medicine should be continued until the viscus returns to its normal condition.

M. *Levy* also has examined this question, and has very ably shewn that intermittent fevers constitute one of the most complex and often obscure class of diseases, giving rise to a multitude of morbid states which are very diverse in their phenomena and effects, but which are all attributable to the same cause, and should all be treated in the same manner. Applying this truly philosophical idea to the development of ascites after agues, he has shewn that this disease is only a consequence, and will, like enlargements of the spleen and other analogous effects of the fever, generally yield to the judicious exhibition of the quinine. . . . . . .

As novel therapeutic suggestions, or as improved and better defined applications of former methods of treatment, we may allude to—1. The experiments and observations of Dr. *Hudson* on the employment of minute doses of the nitrate of silver in some complaints of the digestive mucous membrane; 2, the employment, by M. *Sicherer*, of full doses of calomel in typhus fever; 3, the use of emetics, repeated *coup sur coup*, in the treatment of croup by M. *Jourdain*; 4, of ammonia in diabetes; 5, of sulphur in angina pectoris, &c. &c. As a happy specimen of recurrence to genuine therapeutic analysis, we may cite the memoir of M. *Mascarel* on the treatment of pneumonia in old people by bleeding and large doses of the tartrate of antimony. . . . . .

In toxicology some very valuable researches have been made during the preceding year. We may first cite the experiments of M. *Blacke* on the action of poisons: they tend chiefly to shew that poisonous substances act only by their being brought in contact with the nervous system, after passing through the circulation. The most important results of his inquiries may be stated to be:—

1. The time required for the passage of a poisonous substance through the walls of the capillaries is inappreciable. 2. The interval of time, that elapses between the absorption of a poison by the capillaries and its diffusion throughout the system, cannot exceed nine seconds. 3. There is, however, always a more considerable interval than this between the introduction of a poison into the capillaries or the veins, and the development of the first symptoms; and 4. A poison is found to act proportionately more quickly, the nearer the point at which it is introduced into the circulating system is to the brain.

M. *Orfila* has continued his most interesting researches on different poisonous substances, more especially the salts of arsenic, copper, and antimony, and has beautifully shewn how they penetrate, and may be recovered by chemical analysis from, almost every tissue of the body. Independently of the numerous useful results which this indefatigable experimenter has obtained, our attention should be awake to the comprehensive character of the views which have directed all his labours. The pursuit of the poisonous substance, beyond the theatre to which science had hitherto limited its enquiries, opens a new æra in toxicology; and his multiplied experiments, distinguished as they have been not less by their rigorous exactitude than by their beautiful delicacy, have led him to an order of researches, hitherto entirely novel, on the existence of various heterogeneous substances in the tissues of the body in a state of health.

The third and last article in the Gazette Medicale, on the leading medical events of the year 1840, is devoted to a brief review of the leading new works which have been published during its course. The Essays on General Zoology, by *Isodore St. Hilaire*, the elaborate treatise on General Physiology by *Burdach*, and translated into French by that prince of *traducteurs*, M. *Jourdan* of Paris, the Comparative Anatomy of the Nervous System in its relations with the intellect by M. *Leuret*, and the treatise on Physiology by Professor *Duges* of Montpelier, are mentioned with particular praise.

Among the most valuable publications on pathology, the new edition of M. *Chomel's* treatise is deservedly named, as replete with the soundest views and the most correct principles of practice. Then, too, the new edition of *Andral's* Clinique Medicale is, as might be expected, alluded to in the most flattering terms: it is styled to be "the work, the inspiration, the reflection of the epoch when it was composed, and under this aspect to hold a prominent place in the history of our last medical revolution; it is a work of transition, that is to say, it partakes at the same time of the characters of past times and of the spirit of the present day." The Philosophical treatise of Practical Medicine by M. *Gendrin* (reviewed in the Medico-Chirurgical Review for Oct. 1840) is noticed with some degree of favour. M. *Louis* has brought out a new edition of his treatise on typhoid fever, and has collected fresh proofs of the soundness of his conclusions, that the lesion of the elliptical groupes of the intestinal mucous glands is an invariable attendant of this disease. M. *Forget*, one of the professors of the school at Strasburg, in a work which he has published on follicular enteritis, thus alludes to M. *Louis's* researches :—"Preceding authors had *observed* the lesion in question; MM. *Prost* and *Louis* have *counted* it, and having met with no exception, they have erected, what in the opinion of their predecessors was only an accidental phenomenon, into a fundamental pathological fact: it is to statistics that we owe this magnificent result." M. *Forget* does not neglect to consider the state of the fluids as well as of the solids in typhoid fever, and he has thus blended in his work a Broussaisian Solidism with, what he calls, a rational Humorism . . . . . In the department of surgical pathology and therapeutics, the treatise on External Pathology, by M. *Vidal de Cassis*, deserves to be named as a work remarkable equally for its perspicuity and candour as for its extensive erudition and good arrangement. We must not omit, too, the lectures on Clinical Surgery by that indefatigable writer, M. *Velpeau*, whose merits and whose faults are sufficiently obvious in all his writings.

*Character of M. Velpeau.*—"There can be no doubt that the surgeon of La Charité is not deficient in originality and power of invention, and that, moreover, he possesses a very extended knowledge of every subject that he treats of. But whenever these two qualities are brought prominently forward by an author, we very generally find that one proves injurious to the other. Now this is what occurs to M. *Velpeau.* He aims to pass for very learned; and his lectures, like his writings, and we may add, his practice too, present a living panorama of whatever is new abroad as well as in France. He has been aptly called the *great experimenter of the present time,* applying whatever others propose, and applying it, too, with a certain spirit of modification, if not of improvement. This arises from a disposition in his character to appropriate to himself every suggestion that he at all modifies, and to give a decided preference of his own manner of seeing and doing things to that of the original proposers. Now from this habit, praiseworthy we admit in principle, arise certain inconveniences which it may be worth while to note. M. *Velpeau* often does not make himself sufficient master of every subject that he canvasses and comments upon. Belonging to the school of sentiment rather than to that of experience, he judges of things at once, and from his first impressions, instead of waiting for the results of a complete and rigorous examination. In matters of actual practice he is guided in the same manner. More ruled by the wish to improve than thoroughly to understand, he does not study with sufficient attention the various processes and methods which he applies; he persuades himself that what others have done is neither novel nor important, and whatever change he introduces acquires in his eyes a *brevet* of invention, and a right of personal property. This mode of seeing and doing things gives to the honorable professor a seeming want of gravity and of good faith, and accounts for the numerous accusations which have been brought against him. Perhaps these reflections are applicable rather to the encyclopediacal character of his mind, and of his mode of instruction, than to his teaching itself. Certain it is that, if his lectures aimed less at being so comprehensive, they would gain in accuracy and profundity what they lost in the novelty and variety of their details."

After specifying the titles of numerous works which have been published in France during the course of the past year, the writer closes his remarks by mentioning the names of the most distinguished surgeons and physicians who have died during that period—viz. *Marc, Richerand, Biett, Cloquet, Robiquet, Sedillot, Esquirol,* and *Landré-Bauvais,* members of the Institute; also *Graefe* the eminent surgeon of Berlin, *Omodei,* the well known journalist of *Milan,* Professor *Renchi* of Naples, &c.—*Gazette Medicale.*

---

### APPLICATION OF THE SOLAR AND OXY-HYDROGEN MICROSCOPE TO MINUTE ANATOMY.

M. *Donné,* whose name is doubtless familiar to our readers as one of the most promising physiologists in France at the present time, and who for several years past has devoted much time and study to the microscopical examination of the blood and other animal fluids, has recently addressed the Royal Academy on the applications which he has made of the solar and the oxy-hydrogen microscopes, for the purpose of demonstrating the minute anatomy of the different elements of animal and vegetable structures, the composition of their various juices and fluids, the circulation of the blood and sap, the molecular arrangement of the nervous and muscular tissues, the development of pathological changes, &c.

(There is a now a *Microscopical Society* in London, of which Professor *Owen* is President, and many of the leading naturalists of the day are members. There cannot be a doubt but that much *light* may be thrown on the minute anatomy of healthy and morbid structures by the application of the extraordinarily magnifying powers of the oxy-hydrogen microscope.—*Rev.*)

---

### Case of Gangrena Senilis, with Remarks by M. Roux.

The celebrated surgeon of the Hôtel Dieu objects to the common appellation, the gangrene of old people, given to this disease, on the ground of it not at all indicating the pathological alteration on which it depends. Formerly surgeons attributed this form of gangrene to a direct debility, and a diminished vitality of the parts affected; but the researches of morbid anatomy have shewn that it is the result of a lesion of the arterial system, which occasions the compression of the blood-vessels, followed by a more or less complete obstruction of their tubes. The cause of this obstruction may exist either in the interior of the vessels, or exteriorly to them, or in their parietes.

Another reason for his objecting to the name of the disease is the circumstance of it not being peculiar to old persons, as it is not unfrequently observed in middle-age, and occasionally even in young people.

The development of the gangrene is usually preceded by excruciating pains in the affected part; and in most cases it seems to be spontaneous. In not a few cases, however, some outward injury hastens its explosion and appears to act as the immediate exciting cause. M. *Roux* cited several instances in support of this fact; in some it was a sprain, in others it was a wound or blow, and in others it was the irritation from a mismanaged corn or bunion.

A man, 70 years of age, was recently admitted into the Hôtel Dieu for senile gangrene of the foot, the first symptoms of which had made their appearance some time before. He attributed it to an injury of the nail of the great toe. The gangrene gradually extended upwards to about a third the height of the leg; and there it stopped. M. *Roux*, after waiting a few days, amputated the limb, as he thought that the patient's strength was not sufficient for the protracted process of separation by natural efforts. The anterior tibial artery alone required a ligature; the posterior one was obstructed with a coagulum: the coats of the former were somewhat hard and partook partially of the alteration which the posterior and the other vessels had undergone more completely. Thus the gangrene of the limb was evidently owing to the obliteration of its arteries. Now such a state of the blood-vessels may take place in an acute as well as in a chronic manner, in consequence of an inflammatory condition of their lining membrane.

M. *Roux* alluded to a case of this sort in a young girl, whose arm he was obliged to amputate and who died; and to another case which occurred in a medical man's wife, during the season of the cholera, who died on the very day that the operation was to be performed. But this acute form of the disease is certainly of rare occurrence.—*Gazette des Hôpitaux.*

*Remarks.*—Our chief motive for having introduced these brief observations is to remind our readers of the suggestion of Mr. *Syme* of Edinburgh as to the proper treatment of senile gangrene. Mr. *Syme* objects to the usual practice of giving tonics and stimulants, and points out the great superiority of a lowering and antiphlogistic regimen, "by enforcing a strictly vegetable diet, abstinence from every sort of stimulant, and the maintenance of perfect quiet in the horizontal posture:" vide the last number of the *Medico-Chirurgical Review*, p. 549. (*Rev.*)

---

### NEW METHOD OF COVERING PILLS.

The plan proposed by M. *Garot* for this purpose appears to be very simple, and is said to be very effectual in most instances. It consists in dipping the pills for a moment in a solution of gelatine and allowing this varnish to dry on their surface. The only objection to this method is, that if the pill be soft, or if it contain any oleaginous or oleo-resinous matter, as copaiba balsam, the gelatine by contracting during desiccation firmly around the surface is apt to squeeze this matter out. This inconvenience however is obviated by adding a little gum and sugar to the gelatine that is used. The following is a good mixture for the purpose :—take of dry gelatine *one* part, pate de jujubes *seven* parts, and of water as much as is sufficient. These materials should be slowly dissolved in a sand-bath, so that the mixture may acquire the consistence of syrup. The pills are then picked up with a long needle and dipped in the solution. If they contain any oily or resinous matter, it will be well to give them a second coating afterwards, when they have become quite dry. The little aperture left by the needle should be afterwards closed by touching it with the solution. The mixture which we have recommended, dries almost as quickly as the pure gelatine, and, besides contracting less firmly, it has a pleasant taste in the mouth.—*Journ. des Connaissances.*

### PILLS OF COPAIBA, TURPENTINE, AND CUBEBS.

Dr. *Puche,* of the Hôpital du Midi, recommends the following formula for pills which he has found to be very efficacious in the treatment of gonorrhœa.

Dissolve in a sand-bath some dried (*cuite*) turpentine in an equal quantity of copaiba balsam, and add to the solution as much cubebs powder as may be sufficient to form a consistent mass. The pills or boluses may then be covered with a double coating of the gelatinous mixture mentioned in the preceding article.

### M. RAYER'S FORMULA FOR THE INTERNAL USE OF COD-LIVER OIL.

| | | |
|---|---|---|
| Take of | Cod or skate-liver oil | 90 parts. |
| | Gum Arabic . . . | 10 —— |
| | Water . . . . . . | 60 —— |
| | Syrup of Opium . | 60 —— |

These ingredients are to be well rubbed together, until the mixture is quite smooth and uniform. M. *Rayer* has employed this remedy in numerous cases of chronic pneumonia. He has given the pure oil in several cases of chronic gastritis, when the stomach has been able to bear it: sometimes, however, it is necessary to add a few drops of laudanum to the dose.

### UTILITY OF ALUM IN DISEASES OF MUCOUS MEMBRANES.

The action of alum, as a local application, seems to be extremely beneficial in the various forms of inflammation and ulceration of the different mucous membranes of the body. Thus, in stomatitis or the membranous inflammation of the lining membrane of the mouth, in angina tonsillaris and pharyngea, in some affections of the larynx, in deafness dependent upon an obstruction of the

Eustachian tube, in numerous diseases of the vagina and cervix uteri, &c. this remedy has, when judiciously used, been productive of the greatest advantage.

M. *Delmas*, one of the physicians of the the Montpelier Hospital, has recently published an interesting report of his practice with this agent; and from it we derive the following observations.

It may be unnecessary to allude to various affections of the throat, as the use of alum in the form of a gargle is well known in such cases to all practitioners; but few perhaps are aware of its great utility in some of the forms of ulceration of the generative organs in both sexes. M. *Delmas* has derived great advantage from its employment in those large but superficial ulcerations which are so often found to exist in women affected with leucorrhœa. In the majority of such cases, the neck of the womb is more developed than in health, is very sensibly tumefied, and sometimes presents on its vaginal surface large granulations, which bleed on the slightest contact, and occasionally resemble some form of cancerous ulceration; these are the cases in which the *alleged* cures of cancer of the cervix uteri have been reported to have been effected. M. *Delmas* congratulates himself at having arrived at nearly the same conclusions as M. *Recamier*. This distinguished physician has used alum very extensively in various forms of ulceration of the uterus, and with the happiest results. Although it cannot cure cancer, it is often found to induce an advantageous modification in the diseased action of the adjacent tissues, and thus to retard very materially the progress of the greater evil.

M. *Delmas* has used alum not only in numerous affections of the mucous membranes, but also in the solutions of continuity of the skin. Here the application of it gives rise to much more decided effects, and the pain which it occasions is sometimes so intolerable that it may be necessary to moderate its action by incorporating it with cerate, and by adding, if requisite, some preparation of opium. It is more especially when the ulceration of the skin is connected with a syphilitic condition of the system that we find it expedient to act in this way; for in those old chronic sores, which seem to have acquired a sort of right of domicile in the part, we are seldom obliged to modify the dressing. One of the most remarkable cases, which has occurred in the practice of M. *Delmas*, was observed in a patient affected with elephantiasis. The right leg had become swollen to three times its natural size, and had been for a length of time the seat of an ulceration which occupied the entire lower third at least of the limb. The case had resisted every means that had been tried at different times, and the poor fellow's constitution had in consequence become seriously impaired. After a month's dressing with alum (it is not stated whether it was applied in the form of a powder, or mixed with cerate, or dissolved) more than two thirds of the extensive sore was firmly healed.

.... "In applying alum powder to solutions of continuity of the skin, it is sometimes necessary to mix it with lard, in consequence of the pain it occasions when applied alone. The form of dressing which I most frequently employ, says M. *Delmas*, consists in covering the wound with a thin layer of the powdered alum, and on the following day putting an emollient cataplasm over it, the use of which should be continued until the crust formed by the alum falls off. When this takes place, the surface of the wound is usually found to exhibit a more or less deep red colour, and from this we are to judge whether the application should be repeated or not."

The results of his general experience are summed up in the following conclusions:—

1. That alum acts as a local application in inflammation of mucous membranes by at once counteracting it and causing the morbid process to *abort*.

2. That when applied to solutions of continuity of these membranes, it greatly promotes the healing process, and that its action is never injurious in the hands of a judicious practitioner.

3. That it quickly determines the cicatrisation of ulcerations of the skin; but that its effects in such cases require to be watched, in consequence of the pain and re-action which are sometimes apt to be induced.

4. That in syphilitic ulcerations alum is a powerful local remedy, more especially when the constitutional disease has been already combatted by appropriate general treatment.—*Journal de Med. de Montpelier.*

---

### CASE OF TETANUS CURED BY LARGE DOSES OF MORPHIA, USED INTERNALLY AND ENDERMICALLY.

A young soldier was bitten rather severely in the hand by an insane patient whom he was attempting to restrain. Next day, after being exposed to the heat of a strong sun, he lay down in a field and fell asleep for some hours. In the course of the same evening, he experienced a general *malaise,* accompanied with headache, &c. On the subsequent day he was seized with violent contractions and jerking movements of the muscles of the back and neck, and with rigid stiffness of the limbs, alternating with strong convulsive twitches, so that it required five or six men to keep him in bed. The paroxysms, after lasting for several minutes, gradually subsided; but the intervals between the attacks did not exceed a few minutes. He was bled, and was ordered to take a quarter of a grain of acetate of morphia every half hour; he was then put into a hot bath, and a blister applied between the shoulders, the vesicated surface to be dressed with three quarters of a grain of the morphia, and this to be repeated every four hours. The doses of the medicine were gradually increased; the patient was again bled, and he had 3 or 4 more blisters applied along the back, and repeatedly dressed with morphia. By persevering in this mode of treatment, the symptoms gave way by degrees: and on the fifth or sixth day after the invasion of the attack, the patient was convalescent. He took upwards of 20 grains of the morphia by the mouth, and nearly the same quantity was applied, in the course of five days, to different blistered surfaces.

Within a fortnight after his recovery, he was brought again to the military hospital with a relapse of the convulsive paroxysms: indeed they seemed to be even more severe than on the former attack. So forcible and violent were the contractions of the muscles of the back and neck, that the attendants were afraid that he might either be strangled or break a blood-vessel within the head; for every now and then it was dashed backwards with terrible force. The trunk of the body too, supporting itself at two points on the occiput and on the buttocks, while the spine formed a concave arch, was almost continually bounding upwards with so much violence, as to threaten injury to the viscera within, as well as to the outward parts that were bruised. This attack seemed to have been brought on by intemperance. A similar treatment was followed with like success as on the former occasion.—*Journal des Connoiss. Med. Chirurg.*

*Remark.*—It is not quite correct to call this case one of tetanus; for it is evident that the muscular convulsive contractions were of the *clonic,* and not of the *tonic* kind. We should rather regard it as partaking of the characters of epilepsy and hysteria combined—a complication by no means unfrequent in youths of both sexes.—*Rev.*

